# A lightweight intelligent grading method for lychee anthracnose based on improved YOLOv12

**DOI:** 10.3389/fpls.2025.1688675

**Published:** 2025-12-22

**Authors:** Bing Xu, Zejie Ma, Xueping Su, Xiaoru He, Xianjun Wu

**Affiliations:** School of Computer, Guangdong University of Petrochemical Technology, Maoming, Guangdong, China

**Keywords:** lychee anthracnose, disease classification, YOLOv12, lightweight model, attention mechanism

## Abstract

Anthracnose is one of the primary diseases leading to quality deterioration in lychee. Traditional manual grading methods suffer from low efficiency and high subjectivity. To achieve rapid, non-destructive detection and intelligent grading of lychee anthracnose, while addressing the challenge of balancing high accuracy and lightweight design in detection models, this study proposes a lightweight improved model named LycheeGuard-Lite based on the YOLOv12 framework. By introducing the C3k2_Light module reconstructed with depthwise separable convolutions, a dual-path C2PSA attention mechanism (position-channel dual-path attention), and the wConv2D weighted convolution strategy, the model enhances lesion feature extraction capability while reducing computational complexity.Evaluation was performed on a self-built dataset comprising 14, 576 images of two dominant lychee varieties (‘Feizixiao’ and ‘Baitangying’) collected under multiple lighting conditions and annotated with three severity levels (Mild, Moderate, Severe). The results demonstrate that the model maintains 99.4% mAP50 detection accuracy while reducing its number of parameters to 2.19M (a 12.8% decrease) and computational cost to 4.1 GFLOPs (a 29.3% reduction).This research provides a lightweight and deployable algorithmic foundation for automated lychee disease recognition and intelligent grading, offering practical engineering value for post-harvest fruit sorting and quality management.

## 1 Introduction

Lychee is an economically important fruit in tropical and subtropical regions, with China being the world’s largest producer (contributing approximately 65% of the global yield). Major cultivars such as ‘Feizixiao’ and ‘Baitangying’ are highly susceptible to anthracnose after harvesting, leading to significant economic losses.Anthracnose, primarily caused by the fungus Colletotrichum gloeosporioides, is a destructive postharvest disease that severely impacts fruit quality and market value. Notably, susceptibility to anthracnose varies significantly among different litchi cultivars, highlighting the importance of cultivar-specific disease management strategies (Xu et al., 2025).

**Figure 1:**
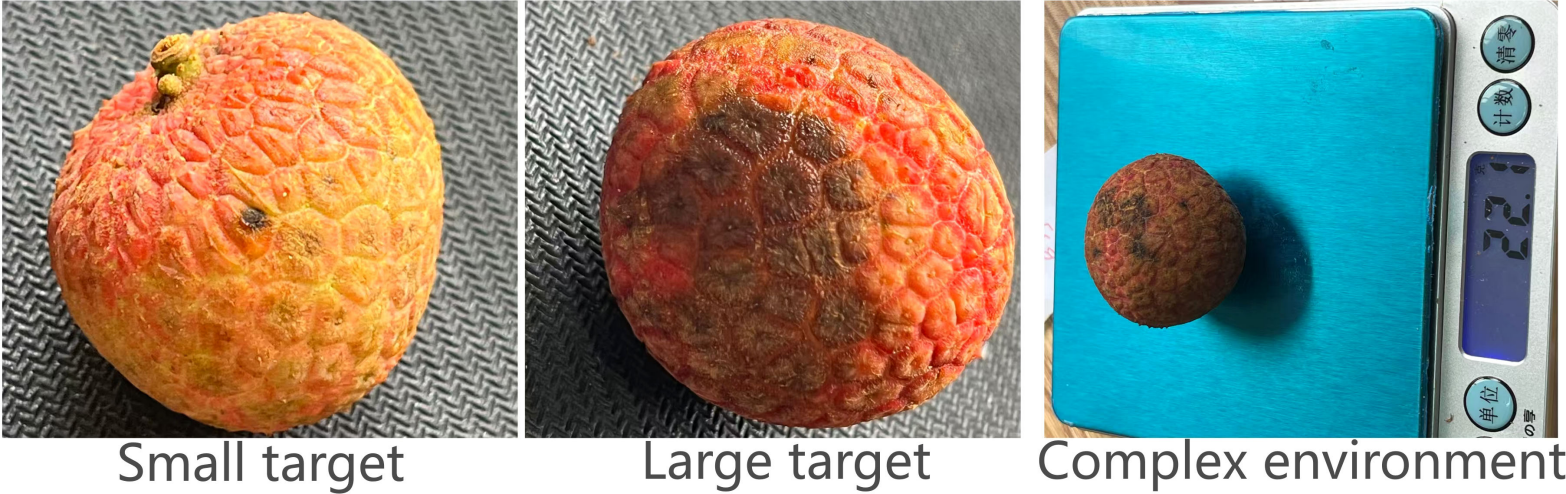
Typical symptoms of anthracnose disease in lychee.

At present, lychee anthracnose grading faces serious challenges. The traditional manual detection method relies on the operator’s experience to judge lesion area and color changes, resulting in low efficiency (about 3–5 seconds/fruit) and poor consistency (with inter-operator grading differences up to 30%), etc. The existing computer vision-based solutions have been successful. Existing computer vision-based solutions, although improved, still have obvious limitations in practical applications: lack of lightweight design hindering mobile deployment, low sensitivity in detecting small-sized spots (≤2mm), and poor stability in complex post-harvest processing environments. Typical manifestations of lychee anthracnose are shown in **Figure 1**.

This study aims to develop a lightweight, accurate, and robust intelligent grading system for lychee anthracnose to address the limitations of existing approaches. A notable gap in the literature is the absence of models specifically designed for the distinctive traits of lychee anthracnose, particularly in detecting small lesions, classifying severity levels (Mild, Moderate, Severe) according to commercial grading standards, and achieving a balance between lightweight architecture and environmental robustness. The core contribution of this work is the proposal of LycheeGuard-Lite, a novel lightweight model based on an enhanced YOLOv12 framework, which incorporates key innovations to bridge these gaps. Several challenges persist in previous studies, such as the difficulty in attaining high recall for small, low-contrast early-stage lesions, the inherent trade-off between model accuracy and computational efficiency that limits deployment on portable devices, and insufficient stability under variable lighting conditions typical of real post-harvest scenarios.

To address the above problems, this study proposes an intelligent grading method based on improved YOLOv12, which achieves a key breakthrough through a triple technological innovation. Firstly, the C3k2_Light lightweight module is constructed to simplify the operation process by adopting a depth-separable convolution structure, which reduces the demand of the model on computational resources at the basic level; secondly, the C2PSA position-channel dual-channel attention mechanism is introduced, which strengthens the ability to capture the key features by optimizing the network structure, and takes into account the effective retention of the feature information while streamlining the parameters of the model;and finally, the wConv2D dynamic convolution optimisation strategy is designed to dynamically adjust the focus of feature extraction based on the spatial distance of the pixels, thereby improving the sensitivity to various types of lesion features in complex scenes.These three improvements form a synergistic effect, not only focusing on solving the bottleneck of the existing scheme in the mobile deployment of the lightweight, but also enhance the detection of small spots, while improving the adaptive stability of the model in the actual post-harvest processing environment, laying a core algorithm foundation for the development of low-cost, portable lychee disease grading and detection equipment, and promoting the development of lychee post-harvest disease detection in the direction of high efficiency, intelligence, and practicality.

## 2 Materials and methods

### 2.1 Related research

Artificial intelligence (AI) technology, especially machine learning, computer vision, and data analysis, is revolutionising agriculture and bringing unprecedented opportunities for agricultural intelligence (Dhanya et al., 2022). The powerful processing capabilities of AI enable rapid and accurate identification and quantification of object features, facilitating effective assessment and grading of agricultural products’ quality to enhance their market value (Dümen et al., 2024; Onmankhong et al., 2024). This automated process not only improves efficiency, but also ensures operational consistency and reduces human error, further enhancing the competitiveness of agricultural products (Silva et al., 2024; Boahen, 2024; Li et al., 2024).

In agricultural visual inspection, there are increasing applications of AI technologies covering various aspects such as crop monitoring systems, crop classification, disease classification and detection (e.g., cassava, maize, potato, grapes, etc.), pest detection, weed detection, and foreign object detection (Vasileiou et al., 2024; Kumar et al., 2025; Taner et al., 2024; Jindal and Kaur, 2024; Cho, 2024; Taspinar et al., 2022). Recent studies continue to push the boundaries in specific agricultural domains, such as the detection of declining trees in poplar plantations (Meng et al., 2025) and the classification of fruit leaf diseases using hierarchical frameworks (Rehman et al., 2023). These applications typically involve classification, segmentation, detection, and counting models with a variety of optimisation algorithms, such as specific convolutional neural network (CNN) architectures, multi-colour spatial fusion, weighted voting, anchorless mechanisms, multi-scale feature fusion, attention mechanisms, and the ViT-CNN double-branching structure (Vasileiou et al., 2024; Chawla et al., 2024; Rodríguez-Abreo et al., 2024; Rasheed et al., 2023). Notably, advanced frameworks like multi-architecture CNNs with MBConv-SE optimization (Sandhi et al., 2025) and multimodal parallel transformers (Zhou et al., 2025) have shown significant promise for complex tasks like fruit disease classification and severity assessment.Research objectives have focused on improving accuracy and efficiency, addressing category imbalance, optimising small target detection, and achieving lightweight designs (Özgür and Saygılı, 2024). The pursuit of lightweight models remains a dominant trend in agricultural AI, driven by the need for deployment on resource-constrained devices (Özgür and Saygılı, 2024). Successful applications are reported in diverse fields, from monitoring maize seedlings using pruned YOLO variants (Jiang et al., 2026) to defect detection in power systems (Wang et al., 2025), underscoring the cross-domain demand for efficient yet accurate models.These studies cover the whole process of visual inspection in agriculture and provide innovative solutions to address challenges such as data scarcity and model generalisation, demonstrating the great potential of AI in agriculture (Chiu et al., 2020; Saadati and Amirmazlaghani, 2024; Gorenshtein et al., 2025; Ribeiro de Oliveira et al., 2023). However, data availability, imaging quality, and the ability to generalise models remain important challenges for the field.Moreover, there is a notable gap in existing research regarding specialized adaptation for “small-spot diseases” (such as lychee anthracnose). However, a notable gap exists in existing research regarding specialized adaptation for “small-spot diseases” such as lychee anthracnose (Vasileiou et al., 2024). These diseases are characterized by small lesion sizes (mild-stage lychee anthracnose lesions are ≤8 mm), low color contrast against the fruit skin (light brown lesions easily blend with the fruit’s texture), and high sensitivity to lighting conditions.While generic solutions for small object detection exist, such as multi-scale feature enhancement in Faster R-CNN (Nazir and Wani, 2025) and algorithmic reviews for UAV imagery (Yan and Zhang, 2025), they are not specifically tailored to the unique challenges of small-spot plant diseases. Currently, most studies focus on diseases with larger lesions (e.g., cassava or corn diseases), with limited optimization for feature extraction precision in small-spot diseases.

As indicated by recent advances in small object detection (Chawla et al., 2024), even with multi-scale fusion strategies, the recall rate for tiny objects (≤10 mm) remains prone to fluctuation. This limitation is particularly pronounced in the detection of lychee anthracnose, making it difficult to directly meet the requirements for accurate grading.

YOLO (You Only Look Once), as a revolutionary single-stage target detection framework, has demonstrated its value in multiple domains with its end-to-end architecture and real-time processing capabilities. The latest version of the YOLOv12 model has demonstrated its potential for application in multiple domains (Su and Zhang, 2023). Particularly in the field of smart agriculture, YOLO models have achieved breakthrough progress. Ranjan Sapkota et al. compared the performance of RF-DETR and YOLOv12 for single-class and multi-class detection of green fruits in complex orchard environments (Sapkota et al., 2025), revealing that YOLOv12 exhibits deficiencies in the recall rate of small-sized fruits (≤10 mm). Manoj Karkee et al. utilized synthetically generated image datasets by large language models to evaluate the performance of the YOLOv12 object detection model for apple detection in commercial orchards, comparing it with previous models and testing its practicality in real orchard images (Sapkota et al., 2025; Sapkota and Karkee, 2025). The results indicated that its accuracy is susceptible to interference under complex lighting conditions. The team of LIANG Xifeng and WEI Zhiwei from China Jiliang University proposed an improved CycleGAN combined with an enhanced NTS-YOLO model to address issues such as low recognition accuracy of tomato stems and branches at night, achieving more precise and efficient segmentation (Liang and Wei, 2025). These agricultural application scenarios analogously reflect the limitations of YOLOv12 in detecting lychee anthracnose:

Firstly, referencing its performance in detecting small-sized fruits, the recall rate of YOLOv12 for mild-stage lychee anthracnose lesions (≤8 mm) may fall short of requirements. Secondly, although the baseline YOLOv12 model possesses a lightweight foundation, there remains room for optimization in mobile deployment. Thirdly, the native architecture has limited adaptability to varying lighting conditions (e.g., front light and back light), which can easily lead to biases in lesion feature extraction.

In the field of agricultural visual recognition, it has become a trend to continuously explore the application boundaries of AI technology (Appalanaidu and KumaraVelan, 2022; Zhang et al., 2024). Among them, lightweight network design is one of the key technological directions (Özgür and Saygılı, 2024). MobileNet is a typical representative of lightweight networks, whose core lies in the depth-separable convolution, which can significantly reduce the number of parameters and computation, making it very suitable for running on mobile devices (París Paricio et al., 2023; Flower MobileNet has been widely used for plant disease detection (Janssens et al., 2020). Building on this foundation, recent efforts like SAM-based augmentation combined with YOLO models (Madadum et al., 2025) demonstrate how advanced data augmentation can further optimize lightweight models for practical agricultural solutions. Another lightweight network is ShuffleNet, which has a channel shuffling operation at its core that reduces computational effort while maintaining feature extraction capabilities and enhancing information exchange between channels (París Paricio et al., 2023).

Two other key technological directions for visual recognition in agriculture are knowledge distillation and model pruning (Özgür and Saygılı, 2024). Knowledge distillation is based on the principle of migrating knowledge from large models to small models, thereby reducing model size and computation without significantly degrading performance (Özgür and Saygılı, 2024). This allows lightweight models to run on resource-constrained devices (e.g., mobile phones, embedded systems) (Özgür and Saygılı, 2024). Model pruning is based on the principle of removing unimportant parts (weights/neurons) of the model in order to compress the model size and increase the efficiency of operation (Özgür and Saygılı, 2024). In agricultural image analysis, model pruning can reduce computational requirements and improve real-time performance.

Data enhancement and transfer learning are two other key technological directions for visual recognition in agriculture (Joshi and Singh, 2020; Sapkota et al., 2024). Data enhancement methods, including rotation, flipping, cropping and colour adjustment, aim to increase the diversity of the training data and simulate images under different conditions (e.g., light, angle, disease stage), thereby improving the generalisation ability and robustness of the model (Chawla et al., 2024; Ahmed et al., 2021). Migration learning approaches, on the other hand, use large-scale datasets (e.g., ImageNet) to pre-train models, which are then fine-tuned on agricultural tasks to significantly improve the performance of the model on a specific task (e.g., plant disease detection) and to reduce the data annotation workload and training time (Mutalib et al., 2024; Rodríguez-Abreo et al., 2024; Unar et al., 2024; Habib et al., 2022). Commonly used pre-trained models include VGG16, InceptionV3 and ResNet50 (Mutalib et al., 2024; Rodríguez-Abreo et al., 2024; Ahmed et al., 2021; Özgür and Saygılı, 2024). However, for lychee anthracnose, general pre-trained models are not adapted to the fruit’s skin texture and lesion characteristics, which may lead to insufficient generalization capability (Mutalib et al., 2024). Therefore, this study requires fine-tuning using a lychee-specific dataset.

The YOLOv12 model demonstrated specific technical advantages in the task of lychee disease classification detection (Su and Zhang, 2023). Its powerful feature extraction network is able to accurately capture disease features (e.g. spot colour distribution, colouring uniformity, blemishes) (Watanabe and Kotani, 2024). The multi-scale feature fusion strategy improves the recognition accuracy of different fruit sizes and local features (Lin et al., 2024). The end-to-end detection architecture drastically reduces the inference time to meet the real-time batch detection requirements (Lin et al., 2024). Lightweight design and optimised loss functions reduce computational complexity while ensuring high accuracy, and are suitable for hierarchical devices or mobile terminals (Lin et al., 2024; Flower et al., 2024). Migration learning and data enhancement enable the model to quickly adapt to the complex texture of lychee surface, lighting changes and shooting angle, showing good generalisation ability (Joshi and Singh, 2020; Lin et al., 2024). However, considering the common challenges in agricultural disease detection (Vasileiou et al., 2024; Özgür and Saygılı, 2024) and the specific characteristics of lychee anthracnose, three major gaps remain in current detection methods.Firstly, most studies only distinguish between “healthy” and “diseased” categories without classifying severity levels (Mild/Moderate/Severe) according to commercial grading requirements, thereby failing to support “high-quality, high-value” workflows.Secondly, there is an absence of a feature enhancement mechanism tailored for small Mild-stage lesions (≤8 mm), making it difficult to achieve consistently high recall rates for such subtle spots (Chawla et al., 2024).Thirdly, lightweight design and environmental robustness have not been jointly optimized. Models tend to either exhibit high parameter counts or suffer from significant accuracy fluctuations under complex lighting conditions, rendering them unsuitable for deployment on portable devices.

Based on the technical advantages of YOLOv12 and the shortcomings of traditional manual methods, this paper proposes the following research objectives and methodological paths. The research objective is to take the YOLOv12 model as the core and carry out progressive optimisation research to improve the accuracy, real-time performance and robustness of the automatic identification and intelligent grading of lychee diseases. The methodology includes continuous improvement of the model structure, optimisation of the data processing strategy, and training with large-scale and diversified lychee image data (covering different diseased spot shapes and disease grades). The ultimate goal is to provide core technical support for the construction of an efficient, accurate and standardised post-harvest processing and quality management system for lychee, and to promote the development of the lychee industry in the direction of intelligence, standardisation and high value.

### 2.2 Data acquisition

#### 2.2.1 Samples and imaging

The research team collected lychee samples of the ‘Feizixiao’ and ‘Baitangying’ varieties from the core production area in Maoming, Guangdong Province, China, during the 2024–2025 harvest season (May–June).High-precision imaging equipment was used to capture images of the three different disease levels of the two market-dominant lychee varieties, namely, Honey-Jar lychee and Concubine-Smile lychee, from different angles. Images were captured from different angles. The photography process was based on natural indoor lighting, and although the background was not completely uniform and simple, it truly reflected the actual state of lychee affected by anthracnose disease. A total of 14, 576 images were collected, providing rich and reliable primary data for the study.

**Figure 2:**
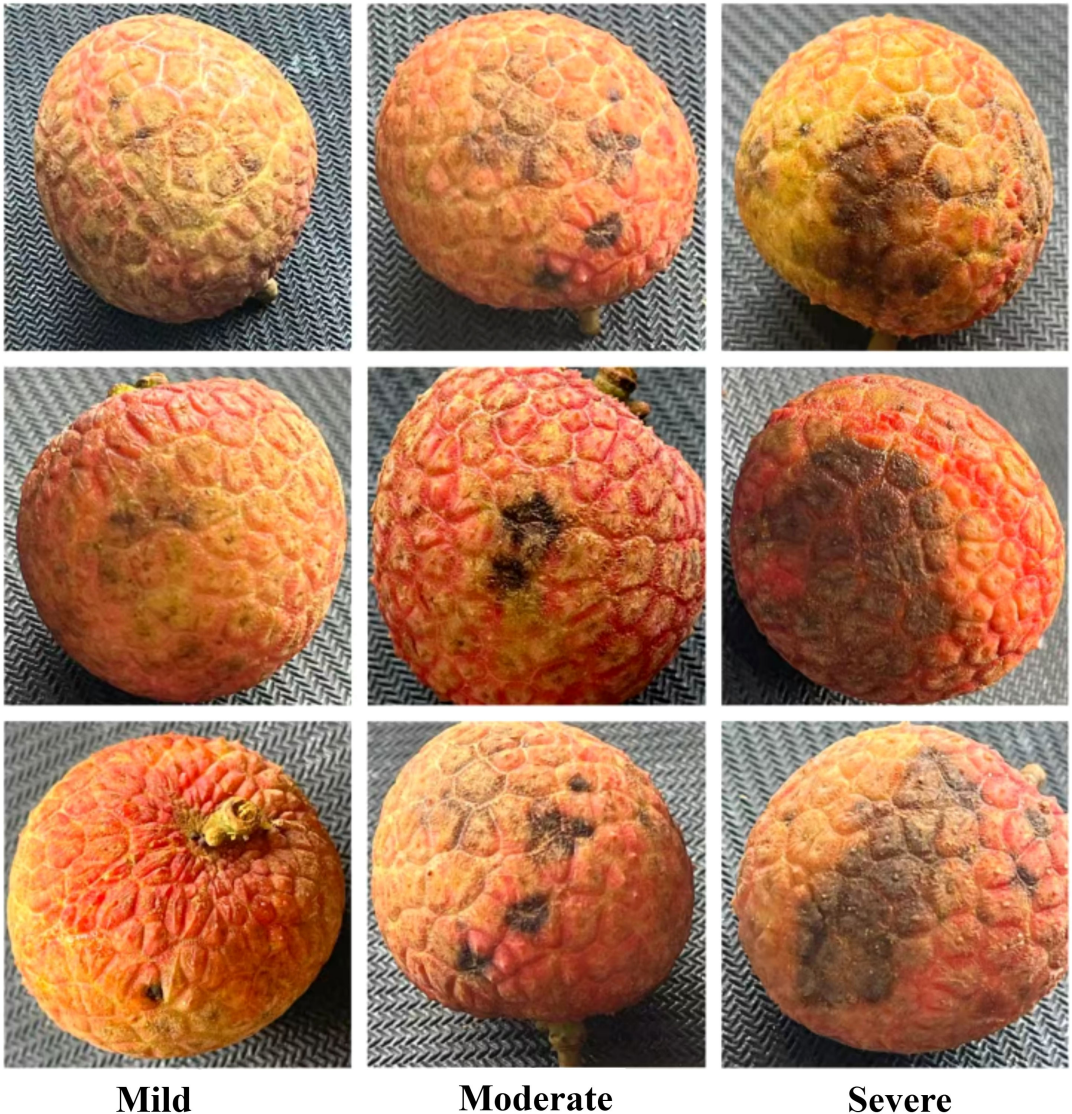
Lychee with three different anthracnose disease levels.

According to the actual needs of lychee commercial treatment, we classified the anthracnose disease of lychee into three grades. The grading standard of anthracnose disease of lychee is shown in **Figure 2**, which is divided into three grades: Mild, Moderate and Severe.

1. **Mild:** the diameter of the lesions is ≤8mm, the colour of the lesions is mostly light brown, only local sporadic distribution, and the impact on the appearance of lychee is relatively small.
2. **Moderate:** the diameter of the lesions is between 8mm < diameter ≤ 20mm, may be accompanied by larger light brown pieces of lesions, and begin to have a more obvious impact on the appearance of lychee.
3. **Severe:** the diameter of the lesions is > 20mm in diameter, dark black or dark brown in colour, widely distributed, seriously affecting the appearance of lychee and may even penetrate deep into the fruit.

To accurately reflect the impact of device diversity in real-world applications, this study selected four mainstream smartphones for image capture. Sensor size, pixel size, and aperture directly affect image signal-to-noise ratio (especially in low-light conditions), depth of field, and detail capture capability. The relevant devices and parameters are shown in **Table 1**. All devices used the main camera (focal length approximately 26mm), with core shooting parameters set to automatic mode: ISO automatically adjusts environmental light sensitivity, white balance (AWB) automatically calibrates colour, and exposure mode (P/Auto) automatically controls brightness.The captured images were directly stored as high-quality JPEG files at the device’s native default resolution, a mode that typically uses pixel binning technology to enhance the signal-to-noise ratio.

**Table 1:** Specifications and default settings of imaging devices used for data acquisition.

Parameter/Model	iPhone 12	Honor 50	Honor X50	realme GT Neo (Speed edition)
Primary Sensor Model	Apple Custom	Samsung HM2 (108MP)	Samsung HM6 (108MP)	Sony IMX682 (64MP)
Effective Resolution	12 MP (Default)	12 MP (9-in-1 binning) 108 MP (Native)	12 MP (9-in-1 binning) 108 MP (Native)	16 MP (4-in-1 binning) 64 MP (Native)
Default Output Resolution	4032 × 3024 px	4000 × 3000 px (binned)	4000 × 3000 px (binned)	4624 × 3468 px (binned)
Native High-Res Mode	Not supported	12032 × 9024 px	12000 × 9000 px	9280 × 6944 px
Sensor Size (inch)	1/2.55”	1/1.52”	1/1.67”	1/1.73”
Pixel Size (μm)	1.4 (Native)	2.1 (binned)	1.92 (binned)	1.6 (binned)
Aperture (f/)	f/1.6	f/1.6	Aperture (f/) f/1.6 f/1.9	f/1.8
Key Features	Smart HDR 3, Deep Fusion	Multi-frame AI Enhancement	Multi-frame Noise Reduction	AI Scene Detection

#### 2.2.2 Data annotation and enhancement

##### 2.2.2.1 Annotation process

**Figure 3:**
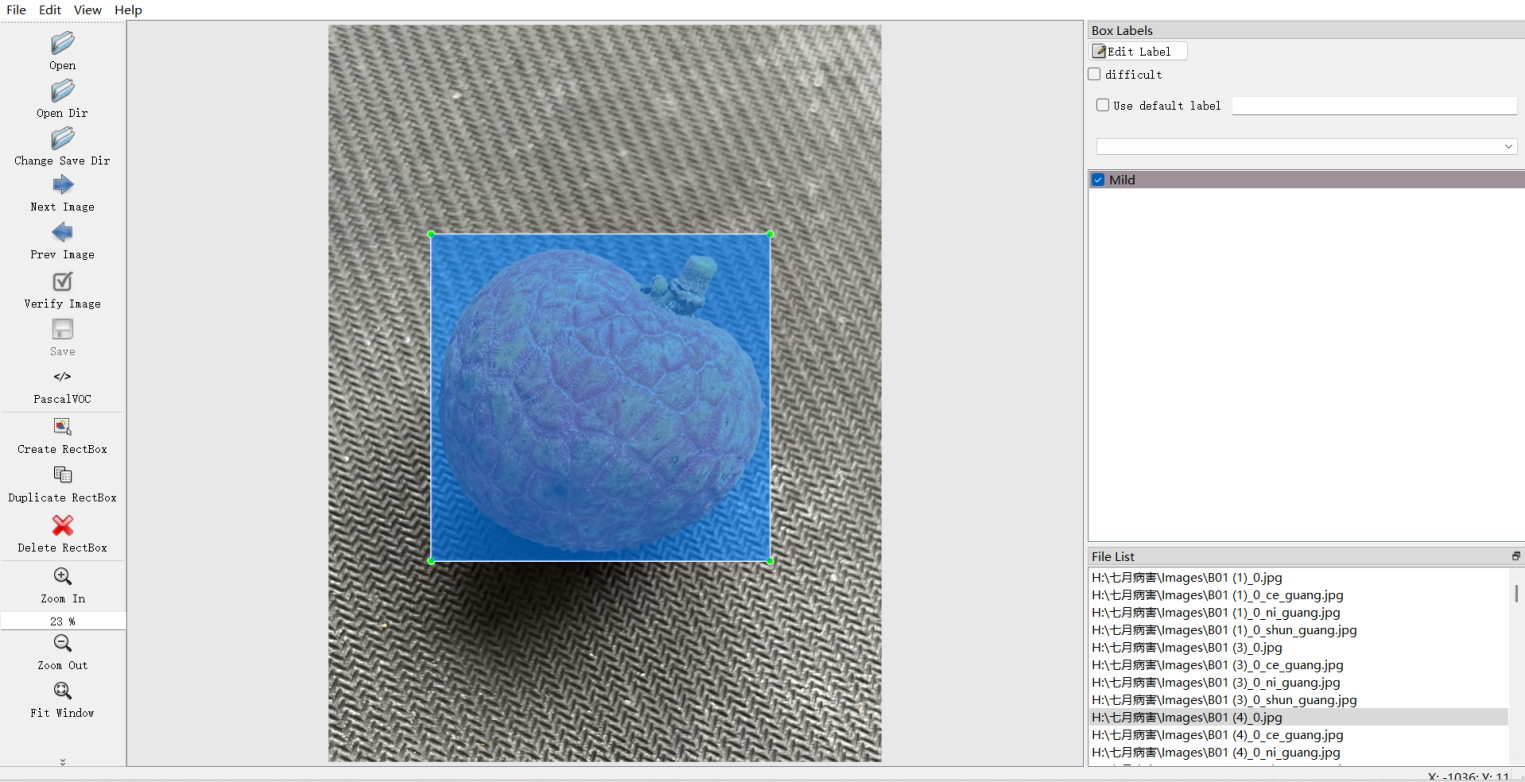
Annotation interfa.

All images were annotated using the LabelImg tool to generate XML files containing object bounding box coordinates and category labels. The annotation process strictly adhered to uniform visual object standards and underwent a three-level quality inspection protocol (including self-check by annotators and review by team leaders) to ensure consistency. In order to adapt to the model training framework, an automated conversion tool was developed to convert the xml annotations to txt files in YOLO format.This strictly regulated annotation and standardized conversion process, through multi-level quality control and automated processing, ensures the accuracy and format standardization of the annotation results from the data source, providing professional and reliable data support for the training and validation of the subsequent lychee disease classification and detection model.Three rigorously trained annotators use the Labelimg tool to precisely outline the boundaries of the lesion areas in the images.All annotation results must be independently verified by at least two experts from the expert group to ensure annotation accuracy. The data annotation diagram is shown in **Figure 3**.

XML is a markup language used for storing and transmitting data, it describes the structure and content of data through custom tags, has good readability and scalability, can flexibly adapt to the data format requirements of different scenarios, widely used in data exchange, configuration files, document markup and other fields, for example, it is often used to annotate the relevant information of the target in the image in computer vision. XML file main content The example is as follows:

**Table:** 

<annotation <folder>Images</folder <filename>B01 (4).jpg</filename <path>… \\B01 (4).jpg</path> <source>… <source <database>Unknown</database> </source <size <width>3024</width> <height>4032</height <depth>3</depth </size <segmented>0</segmented <object <name>Mild</name> <pose>Unspecified</pose> <truncated>0</truncated <difficult>0</difficult> <bndbox> <xmin>561</xmin <xmin>561</xmin> <ymin>1145</ymin> <xmax>2414</xmax <ymax>2933</ymax> </bndbox </object </annotation

The TXT file is used as a text file for YOLO training, storing the target annotation information in the image, and its content is usually recorded in the form of numbers, representing target category, centre x coordinate, centre y coordinate, target width, target height, and the last four items are the normalized values relative to the width and height of the image, which provide the key target positioning and classification basis for YOLO model training.

The main contents of the TXT file are as follows:

**Table:** 

0 0.491898 0.505704 0.612765 0.443452

##### 2.2.2.2 Data enhancement construction

A total of 3644 visible light images of lychee fruits exhibiting anthracnose symptoms were initially collected. To significantly enhance the model’s generalization capability and robustness under complex and variable lighting conditions, particularly addressing the limitation of uniform laboratory lighting, data augmentation techniques were applied to expand the original dataset. The augmentation strategies included adjusting image brightness and contrast to simulate visual effects under different light intensities (e.g., strong sunlight, overcast conditions) and typical lighting angles (e.g., Front light, Side light, Back light). The specific dataset augmentation strategies are detailed in **Table 2**.

**Table 2:** Enhancement strategies.

Enhancement type	Brightness adjustment (magnification)	Contrast adjustment (magnification)	Description of simulation effect
Front light	Brightness is enhanced with an adjustment factor of 1.4	Contrast remains unchanged, with an adjustment factor of 1.0	The visual effect of sufficient light and overall brightness
Side light	Moderate increase in brightness, adjustment factor is 1.2	Significantly increase the contrast, the adjustment factor is 1.3	The visual effect of natural transition between light and dark and rich layers is presented
Back light	Decrease the brightness and adjust the multiplier to 0.6	Contrast is increased and the adjustment factor is 1.2	The visual effect of dark subject and clear edge is presented

The setting of the aforementioned data augmentation parameters (brightness and contrast adjustment multipliers) comprehensively considers the lighting conditions in the actual post-harvest processing environment of lychees and the general image enhancement principles in the field of computer vision. The front lighting parameters (brightness 1.4, contrast 1.0) aim to simulate ample and uniform lighting conditions, moderately increasing brightness to highlight the overall morphology of the fruit while maintaining natural contrast to avoid introducing distortion (Shorten and Khoshgoftaar, 2019). The back lighting parameters (brightness 0.6, contrast 1.2) are used to simulate scenarios where the subject is backlit with distinct contours, reducing brightness to reflect insufficient lighting while significantly enhancing contrast to improve the distinction between diseased spots and the fruit skin edge (Perez and Wang, 2017). The side lighting parameters (brightness 1.2, contrast 1.3) simulate natural light with light and shadow contrasts, aiming to generate images with clear texture and rich layers to enhance the model’s perception ability for subtle surface variations (Buslaev et al., 2020). This parameter combination is designed to cover typical lighting variations in commercial processing, thereby effectively improving the model’s generalization capability and robustness under complex lighting conditions.

**Figure 4:**
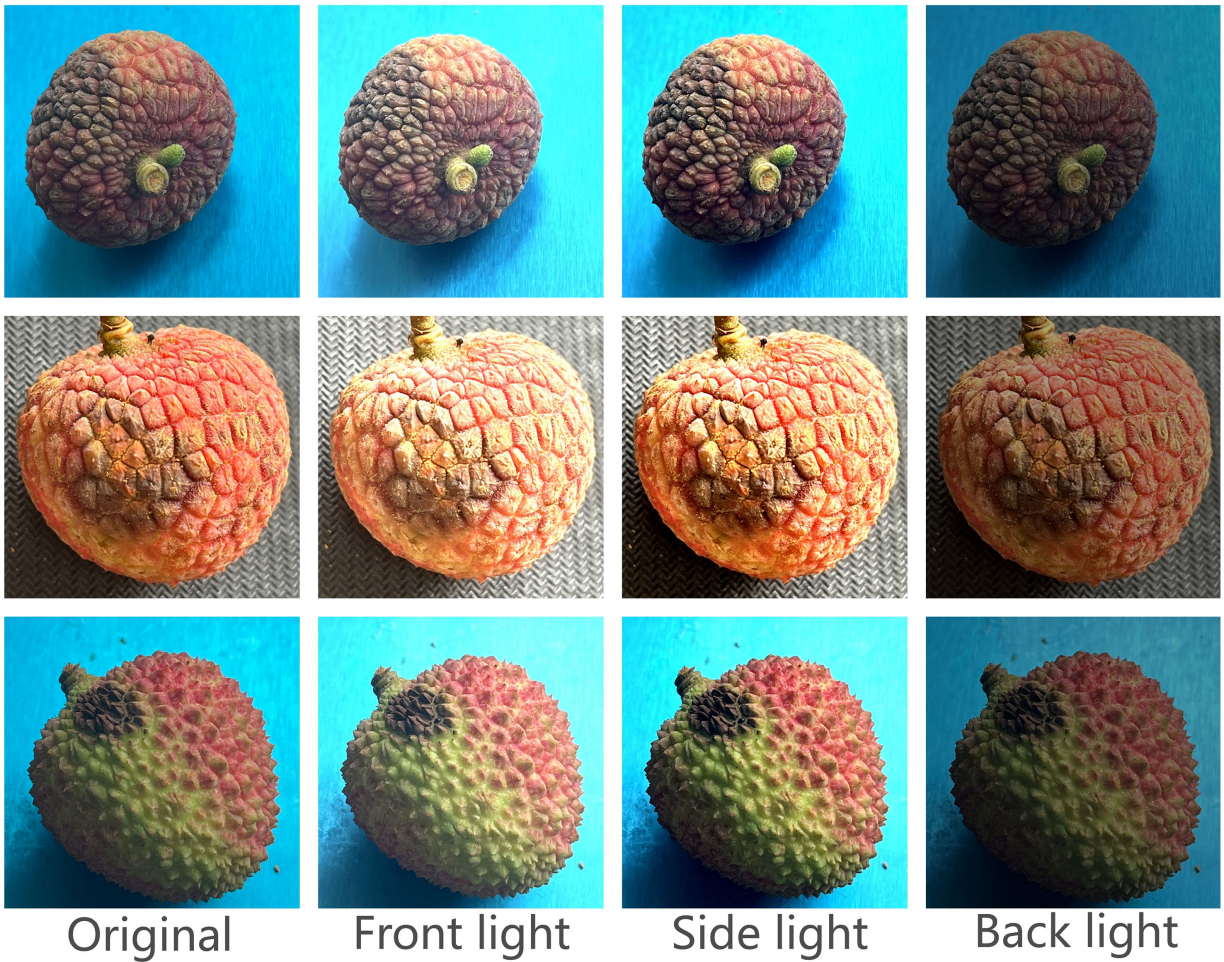
Comparison of data of different enhancement types.

After data enhancement, the total size of the dataset is expanded to 14, 576 images, and the comparison of the effect of the dataset before and after enhancement is shown in **Figure 4**.

There are a total of 14576 images in the enhanced dataset, and the training set, validation set and test set are divided as shown in **Table 3**.

**Table 3:** Dataset partition.

Dataset type	Percentage	Number of images (sheets)
Training Set	70%	10, 203
Validation Set	20%	2, 915
Test Set	10%	1, 458

**Figure 5:**
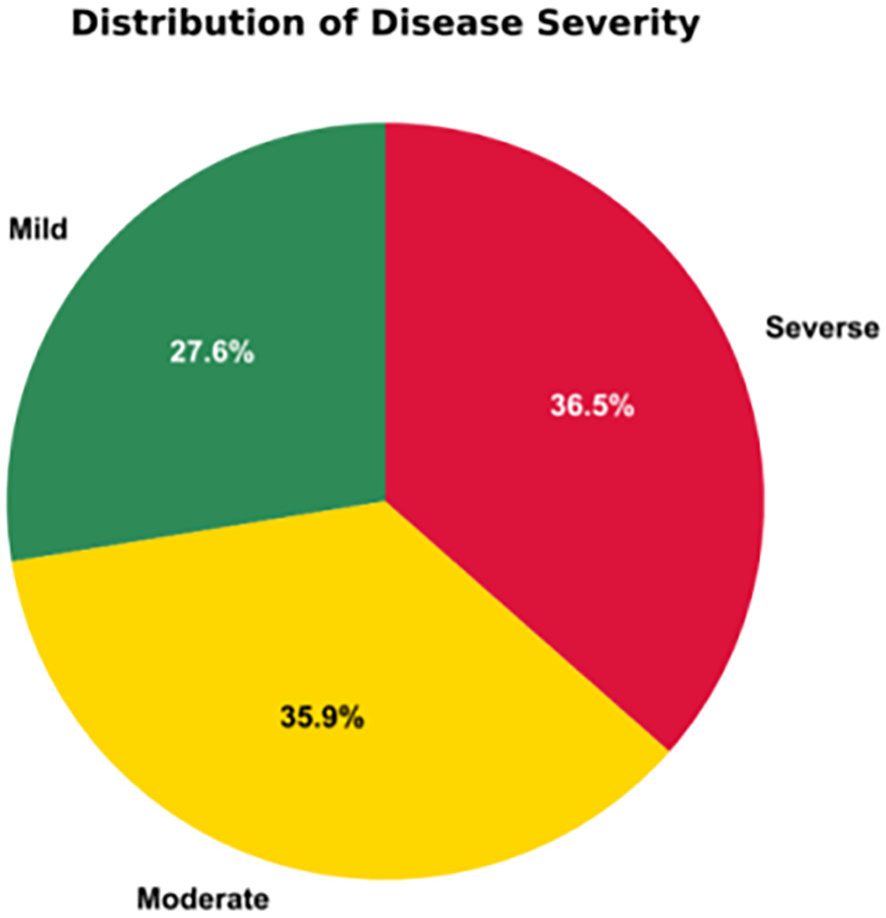
Proportion of images across disease severity levels.

The numbers of Mild, Moderate, and Severe instances are 4, 016, 5, 240, and 5, 320, respectively, and their distribution is illustrated in **Figure 5**.

### 2.3 YOLOv12 algorithm

#### 2.3.1 YOLOv12 baseline architecture

**Figure 6:**
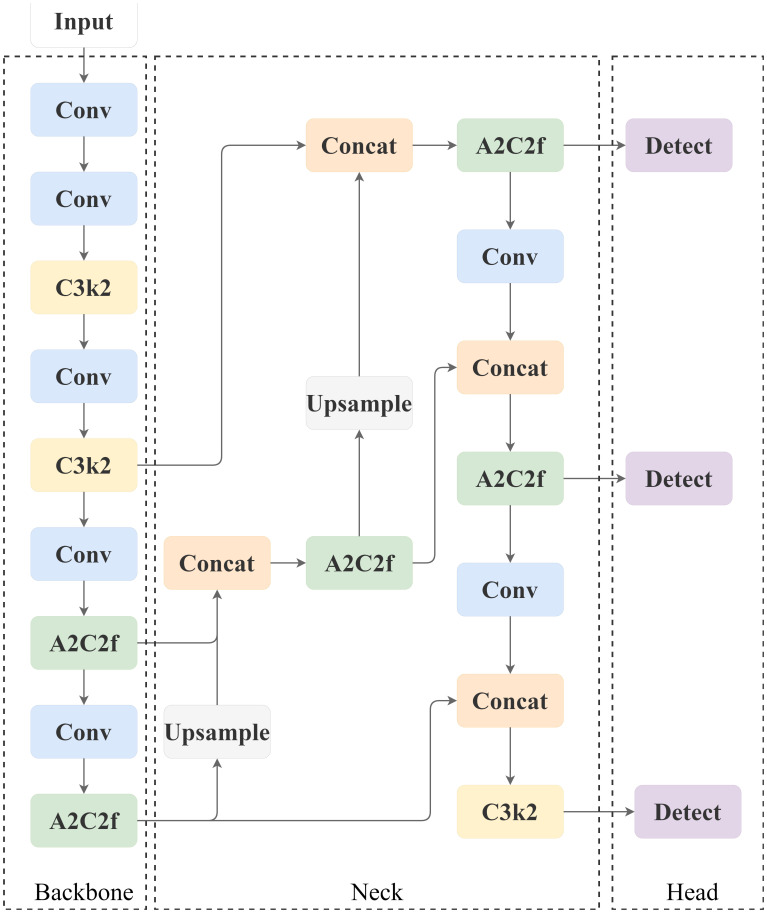
Network structure diagram of YOLO12.

The core architecture of YOLOv12 baseline architecture adheres to the classic design concept of YOLO series, which mainly consists of three parts: Backbone, Neck, Head, and its structure is shown in **Figure 6**.

**(1) Image feature hierarchical extraction architecture (Backbone)**

The feature hierarchy extraction architecture serves as the cornerstone of object detection systems, with its core mission being the construction of a multi-resolution feature pyramid. Through an iterative sequence of feature transformations—incorporating convolutional operators, spatial downsampling operators, and specialized optimization units such as C3k2/A2C2f—this architecture achieves hierarchical compression of spatial resolution and progressive expansion of channel dimensions in feature maps. It thereby systematically encodes visual information hierarchies ranging from pixel-level local patterns to object-level semantic concepts.The final output is a multilevel feature response map (e.g., P3, P4, P5), which characterises the visual patterns in different spatial receptive fields.

① Feature optimisation unit C3k2:

This unit adopts a deep cascade of base transform blocks (Conv-BN-SiLU) as the feature processing engine, and introduces a cross-layer constant mapping mechanism: element-level superposition of the original input with the nonlinearly transformed features. This design achieves multi-scale pattern capture through the layer stacking effect of convolutional kernel, which significantly improves the representation capacity of features; its gradient straight-through path effectively suppresses the signal attenuation in the deep architecture and guarantees the cross-layer integrity of feature information. In deep stacking, the unit drives the exponential growth of the receptive field to achieve the ability to model the complex scene context. Its output discriminative features constitute the input primitives for the subsequent fusion stage.

**Figure 7:**
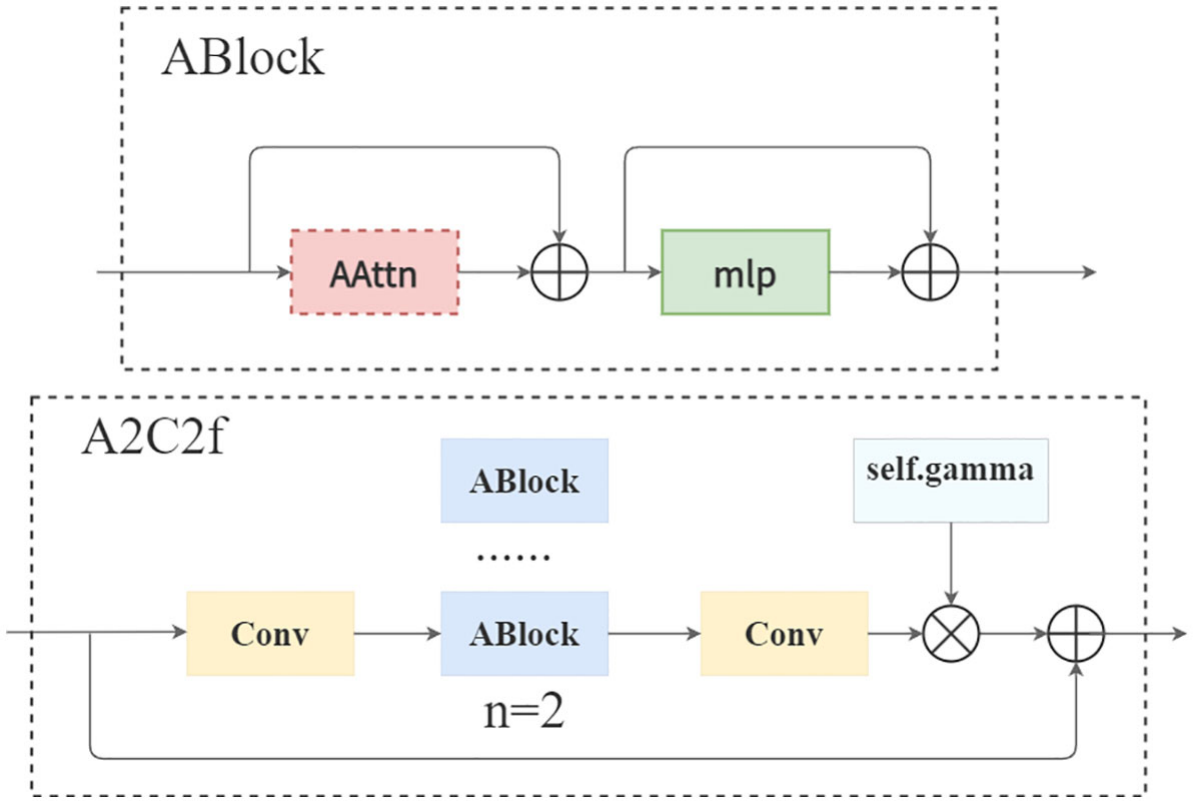
A2C2f model structure.

② Attention Enhancement Unit A2C2f:

The regional attention cross-feature module serves as an innovative feature processor for YOLOv12. This unit improves feature quality under computational efficiency constraints through the collaborative design of regional attention mechanisms and residual structures. Its structure is shown in **Figure 7**.

The core components contain:

① Dimensional projection layer (cv1/cv2), 1×1 convolution to achieve the downscaling/upscaling mapping of feature space.
② Attention computation core (ABlock), fusing regional attention operator and multi-layer perceptron to achieve feature selection and enhancement.
③ Adaptive residual pathway with configurable cross-layer connections to optimise training dynamics and feature robustness.

**(2) Multi-scale feature fusion architecture (Neck)**

Assumes the hub function of cross-layer feature integration. It receives multilevel feature outputs from the backbone network, and establishes bidirectional information flow between features of different abstraction levels through up-sampling operators, channel dimensional splicing operations, and dedicated fusion units (e.g., A2C2f). The cross-scale feature synthesis capability of this architecture complements the fusion of the geometric details of the high-resolution layer with the semantic cues of the low-resolution layer, which significantly enhances the robustness of the model in detecting scale-variant targets (especially tiny targets).

**(3) Target Decoding Output Layer (Head)**

Performs the ultimate decoding of the detection task based on the fused features. A lightweight convolutional network is used to generate the output of three elements: the spatial coordinate parameters of the bounding box, the confidence level of the target’s existence, and the category probability distribution vector. Its design follows real-time constraints and supports dense target prediction at high frame rates.

#### 2.3.2 Lightweight improvements

To enable efficient deployment of the lychee anthracnose detection model on mobile devices, and meet the demand for portability and real-time performance of the detection equipment in the actual post-harvest processing scenario, it needs to be lightweighted and improved. The lightweight improvement mainly revolves around simplifying the network structure, reducing redundant parameters, and optimising the feature extraction process.

##### 2.3.2.1 Basic convolutional variant wConv2D

**Figure 8:**
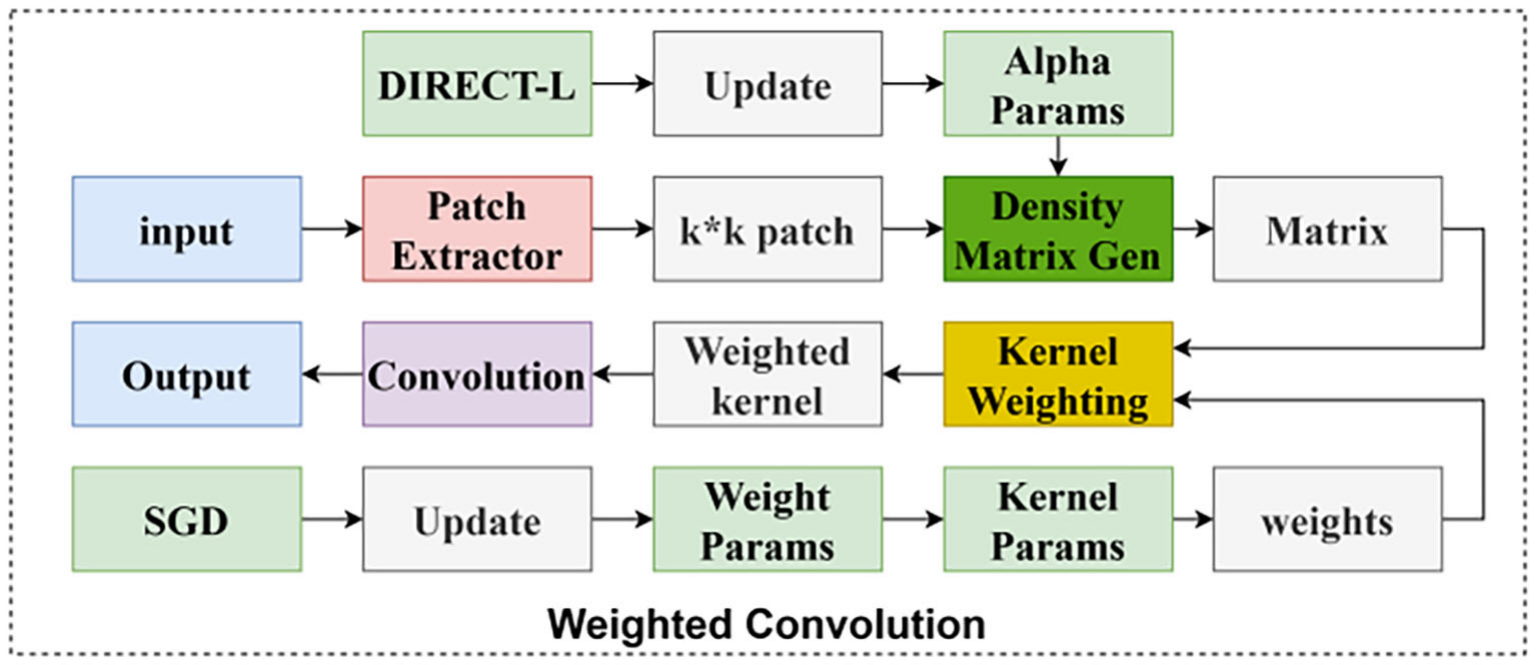
Weighted convolution network structure.

As a base convolution variant, wConv2D is a weighted convolution that adjusts the contribution of neighbouring pixels according to the spatial distance of the pixels through the density function. The density function is optimized by SGD and DIRECT-L algorithms, which can improve the accuracy of lesion detection and achieve model lightweighting. Its structure is shown in **Figure 8**.

**(1) Definition of Weighted Convolution**

Weighted convolution is a novel convolution method for regular grid signals (e.g., 2D images), which scales the contribution of neighbouring pixels by applying an optimal density function, whose degree of contribution is based on the distance of the neighbouring pixels from the centre pixel, which is different from the way of treating all neighbouring pixels equally in the traditional uniform convolution.

**Continuous form: **for the function $\text{f},\text{φ}\in {\text{L}}^{\infty }(\text{Ω})$, the weighted convolution is defined as (Equation 1)

(1) $$\begin{array}{c} \left(\text{f}*{\text{g}}_{\text{φ}}\right)\left(\text{t}\right)\text{ :=}\int_{\text{ Ω}}\text{f}\left(\text{τ}\right)\left(\text{φ}\left(\text{t}-\text{τ}\right)\text{g}\left(\text{t}-\text{τ}\right)\right)\text{dτ} \end{array}$$

Symbol Explanation:

- **f**:Input regular grid signal (specifically referring to 2D lychee image signals here), representing the original data carrier for feature extraction.
- **φ**:a density function that belongs to the essentially bounded function defined on the integral domain Ω, used to dynamically adjust the contribution weight of neighboring pixels to the central pixel based on the pixel spatial distance.
-
${\text{L}}^{\infty }(\text{Ω})$:the essentially bounded function space defined on the integral domain Ω, representing the set of all functions whose values do not exceed a finite value on Ω except for the zero-measure set, ensuring the controllable value range of the density function φ.
- **Ω**:integral domain, the integral domain, corresponding to the spatial coordinate range of the 2D image here, i.e., the rectangular region formed by the row and column coordinates of pixels.
- **t**:spatial coordinate variable of the output feature map, used to locate the position of a pixel in the output feature map.
- **τ**:integral variable, the integral variable, corresponding to the spatial coordinates in the neighborhood of the input signal t, used to traverse the neighborhood pixels related to t in the input signal.
- ***g***:convolution kernel function, the convolution kernel function, used to extract local features of the input signal t, and is the core filtering component in traditional convolution operations.
- **dτ**:differential symbol of the integral variable τ, used to construct the infinitesimal increment unit of the integral operation.

**Discrete form:** the density function is discretised on a 2D regular grid as $\text{Φ}\in {\text{R}}^{{\text{K}}_{\text{a}}\times {\text{K}}_{\text{b}}}$, and the kernel tensor with the density function is defined as ${\text{W}}_{\Phi }\in {\text{R}}^{{\text{K}}_{\text{a}}\times {\text{K}}_{\text{b}}\times \text{F}}$, where ${\left({\text{W}}_{\text{Φ}}\right)}_{\text{ijf}}\;:=\Phi_{\text{ij}}{\text{ω}}_{\text{ij}}^{\text{f}}$, and the discrete weighted convolution is (Equation 2):

(2) $$\begin{array}{c} {\left(I*W_{\Phi }\right)}_{ij}^{f}\text{ :=}\sum_{a=1}^{K_{a}}\sum_{b=1}^{K_{b}}\;\left(\Phi_{ab}\omega_{ab}^{f}\right)I_{i+a-K_{a}+1,j+b-K_{b}+1,.}\\ i=1...R,j=1...C,f=1...F\text{. } \end{array}$$

Symbol Explanation:

- **Φ**:the discrete density matrix, obtained by discretizing the continuous density function φ on a 2D regular grid, with a dimension of ${\text{K}}_{\text{a}}\times {\text{K}}_{\text{b}}$, storing the discretized weight coefficients of neighboring pixels.
-
${\text{R}}^{{\text{K}}_{\text{a}}\times {\text{K}}_{\text{b}}}$:the real matrix space of ${\text{K}}_{\text{a}}$ rows and ${\text{K}}_{\text{b}}$columns, representing the set of all matrices with real elements and dimension ${\text{K}}_{\text{a}}\times {\text{K}}_{\text{b}}$, which is the value space of the discrete density matrix Φ.
-
${\text{K}}_{\text{a}}、{\text{K}}_{\text{b}}$:size of the convolution kernel in the height (a-direction) and width (b-direction), for example, a 3×3 convolution kernel corresponds to ${\text{K}}_{\text{a}}$=3 and ${\text{K}}_{\text{b}}$=3.
-
${\text{W}}_{\text{Φ}}$:the kernel tensor with density function, the core operation unit of discrete weighted convolution, with a dimension of ${\text{K}}_{\text{a}}\times {\text{K}}_{\text{b}}\times \text{F}$, integrating the weight information of the density matrix Φ and the feature extraction capability of the traditional convolution kernel.
-
${\text{R}}^{{\text{K}}_{\text{a}}\times {\text{K}}_{\text{b}}\times \text{F}}$:the real tensor space of ${\text{K}}_{\text{a}}\times {\text{K}}_{\text{b}}\times \text{F}$dimension, representing the set of all tensors with real elements and dimension ${\text{K}}_{\text{a}}\times {\text{K}}_{\text{b}}\times \text{F}$, which is the value space of the kernel tensor ${\text{W}}_{\text{Φ}}$.
- **F**:representing the number of feature channels contained in the feature map generated after the discrete weighted convolution operation.
- **ijf**:i is the row index of the feature map, j is the column index of the feature map, and f is the channel index of the feature map.
- **Φ_ij_**:the element in the i-th row and j-th column of the discrete density matrix Φ, representing the weight coefficient of the neighboring pixel at the corresponding position.
-
${\text{ω}}_{\text{ij}}^{\text{f}}$:the weight of the i-th row and j-th column of the traditional convolution kernel under the f-th channel, the original filtering parameter of the convolution kernel.
- **I**:the input feature map tensor, usually with a dimension of $\text{R}\times \text{C}\times {\text{C}}_{\text{in}}$( ${\text{C}}_{\text{in}}$ is the number of input channels), which is the operation object of discrete weighted convolution.
-
${\text{I}}_{\text{i}+\text{a}-{\text{K}}_{\text{a}}+1,\text{j}+\text{b}-{\text{K}}_{\text{b}}+1,.}$:local pixel block corresponding to the convolution kernel position (a, b) in the input feature map I: $\text{i}+\text{a}-{\text{K}}_{\text{a}}+1$ and $\text{j}+\text{b}-{\text{K}}_{\text{b}}+1$are the starting row and column coordinates of the local pixel block, and “·” means traversing all input channels.
- ***R***, ***C***:height (*R*) and width (*C*) of the input/output feature map, representing the number of pixels in the spatial dimension of the feature map.

Its core advantage is to adaptively adjust the feature extraction weights by pixel spatial distance, which improves the spot detection accuracy while realising the model lightweight.

**(2) Density function optimisation**

Optimisation model: In order to calculate the optimal density function of the learning model, the optimisation model ${\text{min}}_{\text{Φ}}{\text{M}}_{\text{Φ}}$is introduced, where ${\text{M}}_{\text{Φ}}$ is the learning model after replacing the standard convolution with the convolution with density function, i.e. (Equation 3)

(3) $$\begin{array}{c} \;{ℳ}_{\text{Φ}}{\text{: }}_{\text{W}}^{min}ℒ\left(T,\hat{T}\left({\text{W}}_{\text{Φ}}\right)\right) \end{array}$$

Symbol Explanation:

- **min_Φ_**:minimization operator with respect to the discrete density matrix Φ, representing finding the parameter value that optimizes the performance (minimizes the loss) of the learning model ${\text{M}}_{\text{Φ}}$ within the feasible domain of Φ.
**·M_Φ_**: learning model after replacing standard convolution, the LycheeGuard-Lite sub-model that uses the convolution kernel ${\text{W}}_{\text{Φ}}$ with density function instead of the traditional convolution kernel, used to learn the feature patterns of lychee anthracnose lesions.
- **W**:set of all trainable parameters of the learning model ${\text{M}}_{\text{Φ}}$, in addition to the kernel tensorit ${\text{W}}_{\text{Φ}}$also includes other convolution layer weights, fully connected layer parameters, etc., in the network.
- **L**:loss function, used to measure the difference between the model prediction results and the real labels, and the combined loss of CIoU loss and cross-entropy loss commonly used in target detection tasks is adopted here.
- **T**:training dataset, containing image samples labeled with lychee anthracnose severity (Mild/Moderate/Severe) and corresponding bounding box labels.
-
$\hat{\text{T}}({\text{W}}_{\text{Φ}})$:set of model prediction results based on the kernel tensor ${\text{W}}_{\text{Φ}}$, including the predicted bounding box coordinates, category probabilities, and confidence.

Properties of the density function: it is symmetric, α=β, α(i)=α(K−i+1); the central node values are ${\text{α}}_{\text{m}}=\text{M}$ and $\text{Φ}={\text{αα}}^{⊺}$, which are symmetric, semi-positive definite matrices of rank 1, defined by coefficients.

Symbol Explanation:

- **α, β**:1D symmetric coefficients of the density function, corresponding to the weight distribution coefficients of the convolution kernel in the height and width directions respectively, and α=β means that the weight distribution of the density function is symmetric in the two directions.
- **i**:index of the coefficient α, representing the position of the coefficient in the 1D sequence.
- **K**:size of the convolution kernel, the size in the height or width direction (since α=β, the sizes in the two directions are the same).
- **α_m_**:central node coefficient of the density function, m is the index of the central node (for a K×K convolution kernel, m=(K + 1)/2 when K is odd).
- **M**:value of the central node coefficient, the coefficient with the largest weight in the density function, ensuring the leading role of the central pixel in feature extraction.
- **α^T^**:transpose of the coefficient α, converting the 1D coefficient sequence into a column vector.
- **Φ=αα^T^**:rank-1 decomposition form of the discrete density matrix Φ, “**·**” denotes matrix multiplication, and this form ensures that Φ is a symmetric positive semi-definite matrix.

Optimisation algorithms: the kernel weights are optimised using stochastic gradient descent (SGD), a local method for differentiable functions that minimises the loss function by updating the parameters along the negative gradient direction, and the density function is optimised using the DIRECT-L algorithm, a global optimisation method for optimising non-differentiable functions. These optimisation algorithms can efficiently find the optimal density function, further reducing the computational and parametric quantities of the model.

##### 2.3.2.2 C3k2_light

**Figure 9:**
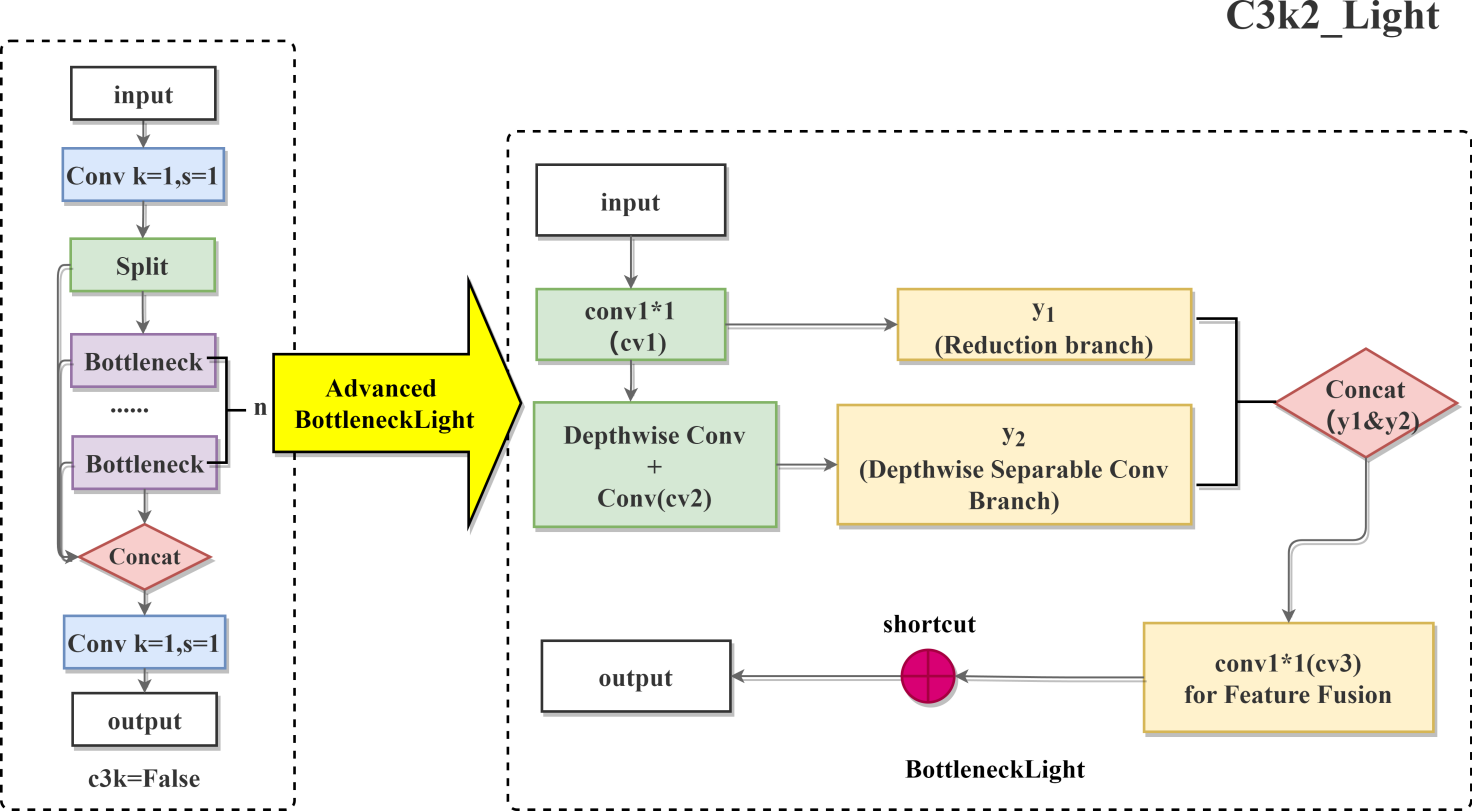
C3k2_Light composite structure.

In order to balance the feature extraction capability and computer efficiency, this study proposes the BottleneckLight module and constructs the C3k2_Light composite structure, the original C3k2 structure is shown in **Figure 9**. Although the original C3k2 module can accurately distinguish different disease levels of lychee anthracnose and provide key technical support for its efficient non-destructive detection and accurate grading, it suffers from a large number of parameters, high computational overhead, and insufficient lightweight design, which makes it difficult to adapt to the actual demand of rapid detection on the mobile terminal.

**(1) Module composition and calculation optimisation**

Input/output channels: inherit standard bottleneck module design (input channel c1, output channel c2).
Intermediate channel: c_ = int(c2 * e) (expansion factor e controls dimensional compression rate).

Convolutional layer reconstruction:

cv1: 1×1 convolutional dimensionality reduction (c1→ c_).
cv2: Depth-separable convolution set (Depthwise Conv 3×3 + Pointwise Conv 1×1), keeping the number of channels c_.
cv3: 1×1 convolutional fusion splicing features (input channel 2×c_, output channel c2)

Residual connection: activated when shortcut=True and c1=c2.

**(2) Forward propagation process**

① Primary feature compression (Equation 4)

(4) $$\begin{array}{c} {\text{y}}_{1}=Conv1\left(\text{x}\right) \end{array}$$

Symbol Explanation:

- **y_1_**:feature map after dimension compression, the output of the “dimension reduction branch” in the C3k2_Light module, retaining the global information of the input features.
- **Conv1**:1×1 convolution operation, its core role is to compress the number of input channels and reduce the subsequent computation.
- **x**:input feature map of the C3k2_Light module, with a dimension of $H\times W\times c_{1}$(*H* is the height of the feature map, *W* is the width of the feature map, and *c*_1_ is the number of input channels).
- ***c*_1_**:number of channels of the input feature map
- *c*:number of intermediate channels, calculated by the formula $c=\mathrm{int}(c_{2}\times e)$, where int(·)is the rounding function, *c*_2_is the number of output channels of the module, and *e* is the channel expansion factor (usually 0.5).

Compress the input channel from c1 to c_(c_=int(c2*e)) by 1×1 convolution, reduce the amount of subsequent calculations, achieve: self.cv1 = Conv(c1, c_, kernel=1).

② depth feature extraction (Equation 5)

(5) $$\begin{array}{c} {\text{ y}}_{2}=PointwiseConv\left(DepthwiseConv\left({\text{y}}_{1}\right)\right) \end{array}$$

Symbol Explanation:

- **y_2_**:feature map after depthwise feature extraction, the output of the “depthwise separable convolution branch” in the C3k2_Light module, enhancing the local detail information of the input features.
- **DepthwiseConv**: 3×3 group convolution (number of groups=c_) to extract spatial features, the amount of computation is only 1/9 of the standard convolution.
- **PointwiseConv**: 1×1 convolution to maintain the number of channels, in preparation for the subsequent splicing.

③ Two-way feature fusion (Equation 6)

(6) $$\begin{array}{c} {\text{y}}_{concat}=concat\left({\text{y}}_{1},{\text{y}}_{2}\right) \end{array}$$

Symbol Explanation:

- **y_concat_**:feature map after two-way feature concatenation, integrating the global information of the “dimension reduction branch” and the local information of the “depthwise separable convolution branch”.
**·concat()**:channel-wise concatenation operation, superimposing y_1_and y_2_ in the channel dimension, and the number of channels after concatenation is the sum of the number of channels of y_1_ and y_2_.(i.e., c_+c_=2c_).

Splicing the original features after dimensionality reduction y_1_ (retaining global information) with deep features y_2_ (enhancing local details), the number of channels is extended to 2×c_.

④ Channel relabelling (Equation 7)

(7) $$\begin{array}{c} {\text{y}}_{\text{fuse}}=\text{Conv}3({\text{y}}_{\text{concat}}) \end{array}$$

Symbol Explanation:

- **y_fuse_**:feature map after channel relabelling, completing the final reorganization of features, so that the number of channels matches the output requirements of the module.
- **Conv3**:1×1 convolution operation1×1 convolution operation its core role is to compress the concatenated 2c_ channels to the target output channel number c_2_, realizing the channel dimension standardization of features.
- **c_2_**:number of output channels of the C3k2_Light module, usually equal to the input channel number c_1_(to facilitate the activation of residual connections).

Adjust the number of channels from 2×c_ to the target output c2 by 1×1 convolution to complete the feature restructuring.

⑤ Residual reorganisation (Equation 8)

(8) $$\text{y}=\{\begin{array}{c} \text{x}+{\text{y}}_{\text{fuse}}\;\;\;\;\;\;\text{if add}\\ \;{\text{y}}_{\text{fuse}}\;\;\;\;\;\;\text{otherwise} \end{array}$$

Symbol Explanation:

- **y**:final output feature map of the C3k2_Light module, the result after residual reorganization, balancing feature extraction capability and training stability.
- **add**:condition for activating residual connection, when *s*h*ortcut*=*True* (the module is configured to enable residual) and *c*_1_=*c*_2_(the number of input and output channels is equal), add is true, otherwise false.
**·x+y_fuse_**:residual connection operation performing element-wise addition between the module input feature map x and the channel-relabelled feature map ${\text{y}}_{\text{fuse}}$, alleviating the gradient vanishing problem in deep networks.
- **otherwise**:case where residual connection is disabled, in this case, the module only outputs the channel-relabelled feature map ${\text{ y}}_{\text{fuse}}$.

**(3) Structural advantages (compare with labelling C3k2)**

① Lightweight convolution: depth separable convolution replaces standard 3×3 convolution, reducing parameter redundancy and achieving computational compression.
② Feature interaction enhancement: dual-path design improves lesion detail retention ability.

##### 2.3.2.3 C2PSA

**Figure 10:**
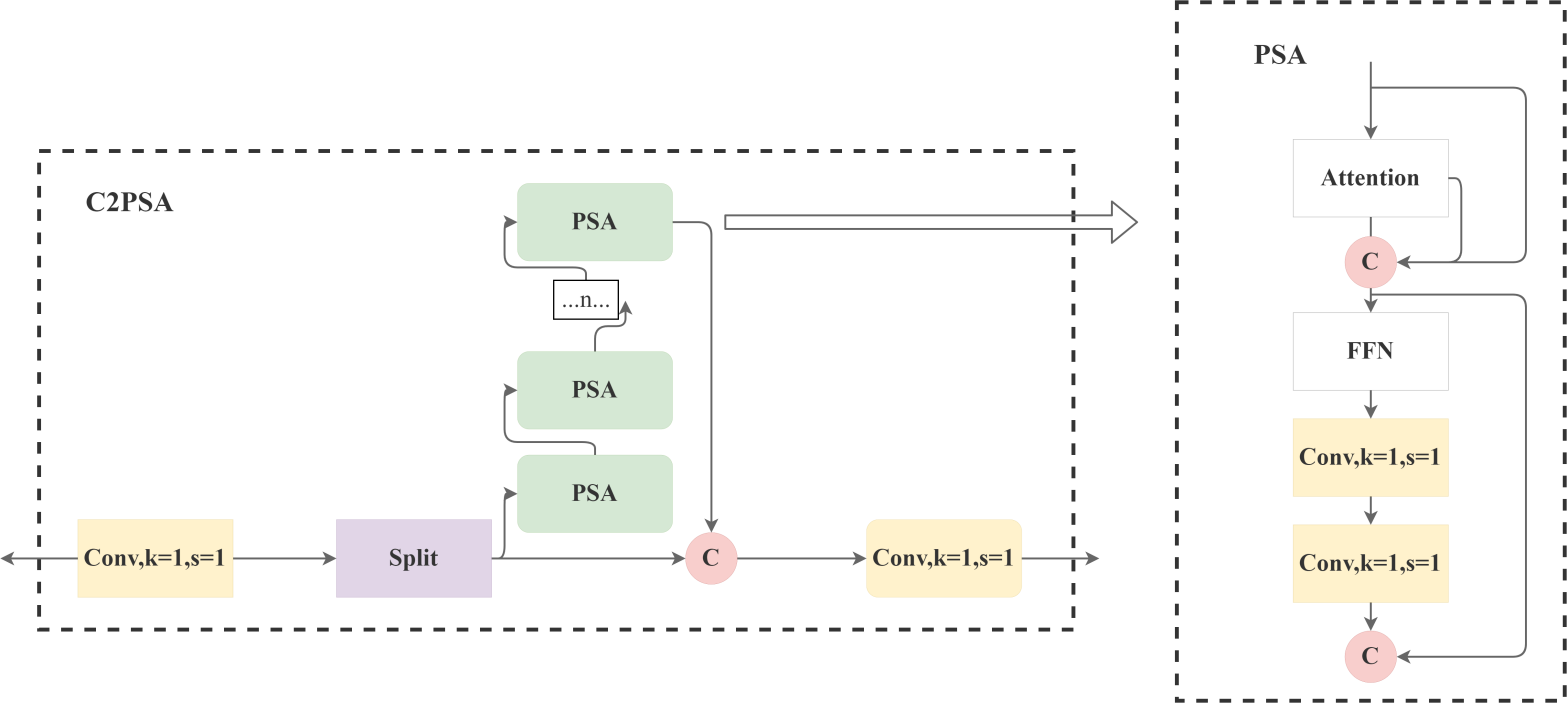
C2PSA network architecture diagram.

The C2PSA module focuses on solving the problem of missing shallow details in deep feature maps, and strengthens the multi-scale characterisation capability through cross-layer feature fusion. Its structure is shown in **Figure 10**.

Feature fusion mechanism:

P3'=Conv1×1(P3))

P4'=U2×(Conv1×1(P4))


P5'=D2×(Conv1×1(P5))

Symbol Explanation (Equation 9):

(9) $${\text{P}}_{\text{out}}{\text{=Conv}}_{3\times 3}(\text{Concat}[\text{P}3^{\prime },\text{P}4^{\prime },\text{P}5^{\prime }])$$

- **Basic feature inputs**:P_3_(shallow layer, 52×52×256), P_4_(middle layer, 26×26×521), and P_5_(deep layer, 13×13×1024).
- **Conv_1×1_**:1×1 convolution operation( Y=W*X+b) that unifies the channel count of the three feature types to 256, reducing computational complexity while retaining core information.
- **U_2×_**:2× bilinear upsampling that upsamples the resolution of P_4_ to 52×52, aligning it with P_3_’;P_5_’ is obtained by two consecutive 2× upsampling of P_5_ to ensure consistent spatial scale.
- **Conv_3×3_**:3×3 convolution( $Y={\text{W}}_{3\times 3}*\text{X}+\text{b}$)that compresses the concatenated features to 256 channels, captures local correlations, and outputs the enhanced feature ${\text{P}}_{\text{out}}$, balancing lightweight design and detection performance.

Firstly, for the feature maps from different layers of the backbone network (P3, P4, P5), a 1×1 convolution is applied to unify the number of their channels to reduce the subsequent computational complexity. Then, a 2-fold upsampling (U_2x_) operation is performed on P4, and a 2-fold downsampling (D_2x_) operation is performed on P5, so as to make them consistent with the resolution of P3, and to avoid redundant feature computation. Subsequently, the processed P3’, P4’, and P5’ are spliced (Concat). Finally, the 3×3 convolution (Conv_3x3_) used to fuse the spliced features and output the enhanced feature map P_out. This mechanism can effectively reduce the number of parameters while integrating shallow high-resolution texture information (which is helpful to capture light-coloured spot features) and deep rich semantic information (which supports spot classification).

**Figure 11:**
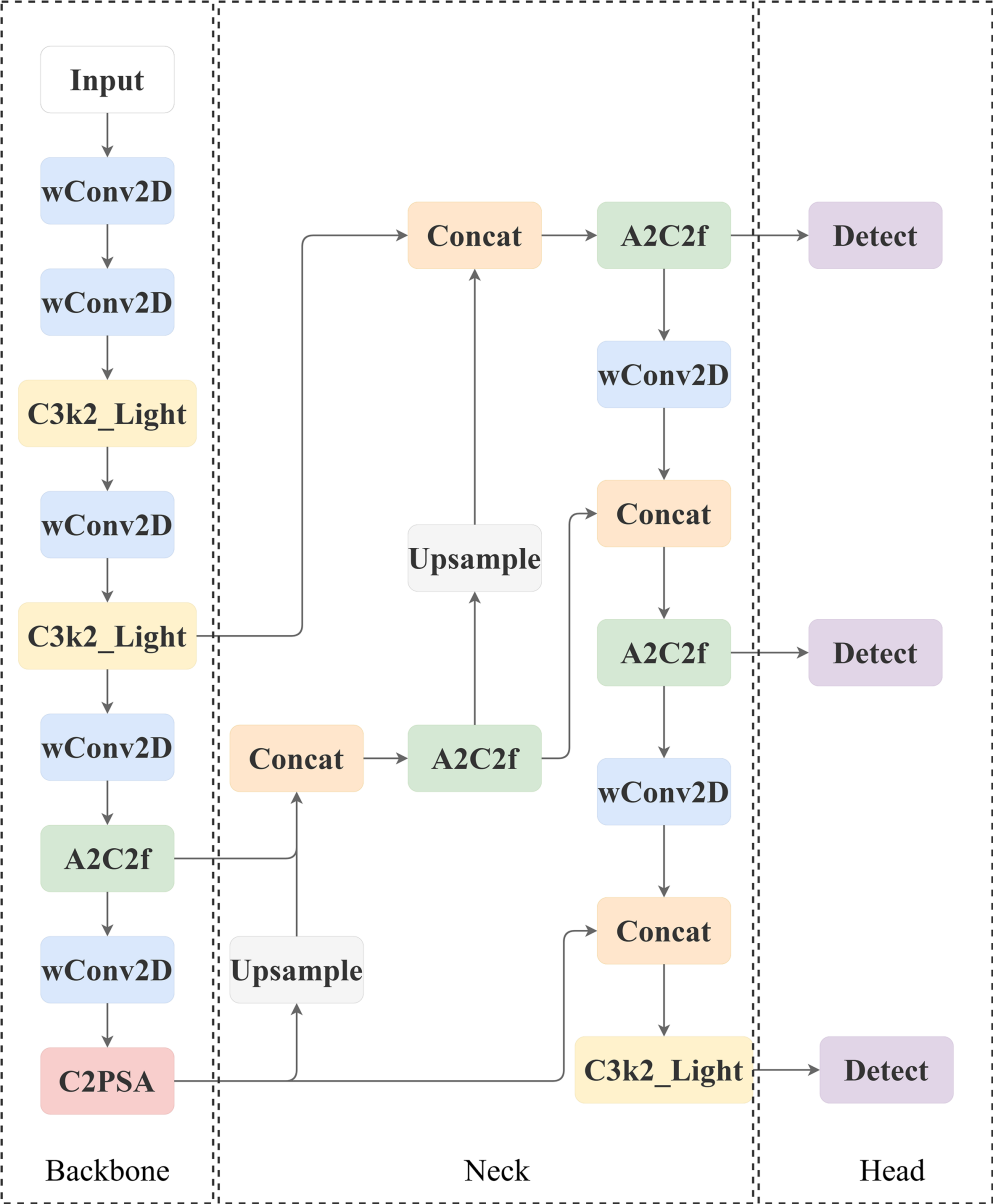
LycheeGuard-Lite network structure.

The improved LycheeGuard-Lite network result graph is shown in **Figure 11**:

### 2.4 Experimental setup

To ensure the reproducibility of our experiments and provide a clear foundation for the results discussion, this section details the experimental setup, including the hardware and software environment, dataset partitioning, training parameters, and evaluation metrics.

#### 2.4.1 Hardware and software environment

All experiments, including model training, validation, and testing, were conducted on a workstation equipped with an NVIDIA GeForce RTX 3060 graphics card (12GB VRAM). The software environment was based on the CUDA 12.6 parallel computing platform and the Python 3.10.18 programming language. The deep learning framework utilized was PyTorch, and the model was developed and trained within the YOLOv12 codebase.

#### 2.4.2 Dataset partitioning

The enhanced dataset, totaling 14, 576 images, was randomly divided into training, validation, and test sets to facilitate model development and unbiased evaluation. The specific partition ratios and corresponding image counts are summarized in **Table 3** (as presented in Section 2.2.2). This partitioning strategy ensures that the model is trained on a substantial portion of the data, tuned on a separate validation set, and its final performance is reported on a held-out test set that was never used during training or hyperparameter adjustment.

#### 2.4.3 Training configuration and hyperparameters

The model was trained for 200 epochs to ensure sufficient convergence. The training process employed the Stochastic Gradient Descent (SGD) optimizer with an initial learning rate (lr0) of 0.01, a momentum of 0.937, and a batch size of 32. The input image size (imgsz) was uniformly resized to 416×416 pixels throughout the training and inference phases. This combination of hyperparameters was chosen to balance training efficiency and model generalization capability. A comprehensive list of the core training parameter settings is provided in **Table 4**.

**Table 4:** Training parameter setting.

Parameter	Setting
imgsz	416*416
epochs	200
batch	32
true	true
momentum	0.937
lr0	0.01
optimizer	SGD

#### 2.4.4 Evaluation metrics

A rigorous quantitative evaluation framework was established using four core metrics common in object detection tasks: Precision (P), Recall (R), mean Average Precision at an IoU threshold of 0.5 (mAP50), and mean Average Precision over IoU thresholds from 0.5 to 0.95 (mAP50-95). Their mathematical definitions are provided in Section 3.1. These metrics collectively assess the model’s detection confidence, its ability to identify all true lesions, and its localization accuracy across different levels of stringency.

## 3 Results

### 3.1 Evaluation metrics

In the field of computer vision, the performance evaluation of target detection models needs to establish strict quantitative standards. In this study, four core metrics are used to construct the evaluation framework: Precision (P), Recall (R), mean Average Precision (mAP), and cross-threshold Average Precision (mAP50-95), which are defined based on the following theoretical foundation:

①Precision (Precision, P) characterises the confidence level of the positive prediction result of the model, and the mathematical expression is (Equation 10):

(10) $$\text{P=}\frac{\text{TP}}{\text{TP+FP}}$$

Where, TP true positive sample number (correctly detected target instances), FP (False Positive) is the number of false positive samples (wrongly detected background areas).

② Recall (R):Reflects the proportion of samples in the true positive category that are correctly predicted by the model, calculated as (Equation 11):

(11) $$\begin{array}{c} \text{R}=\frac{TP}{TP+FN} \end{array}$$

FN: Number of false negative samples (true targets of missed detection)

③ Mean Average Precision (mAP): first the single-category mean average precision (AP), i.e., the area under the precision-recall curve (PR curve), is calculated (Equation 12):

(12) $$\text{AP}=\int_{0}^{1}\text{P}(\text{R})\text{dR}$$

Subsequently, the AP of multiple categories is averaged to obtain mAP (Equation 13):

(13) $$\begin{array}{c} mAP=\frac{\sum_{\text{i}=1}^{\text{N}}AP_{\text{i}}}{\text{N}} \end{array}$$

Where N is the total number of detection categories.

④ mAP50-95: the model robustness is assessed by intersection and merger ratio (IoU) threshold spatial sampling, which is uniformly sampled in the interval of IoU ∈ [0.5, 0.95] in steps of Δ=0.05, and comprehensively evaluates the model performance under different positioning accuracy requirements.

### 3.2 YOLOv12 baseline model performance evaluation

**Figure 12:**
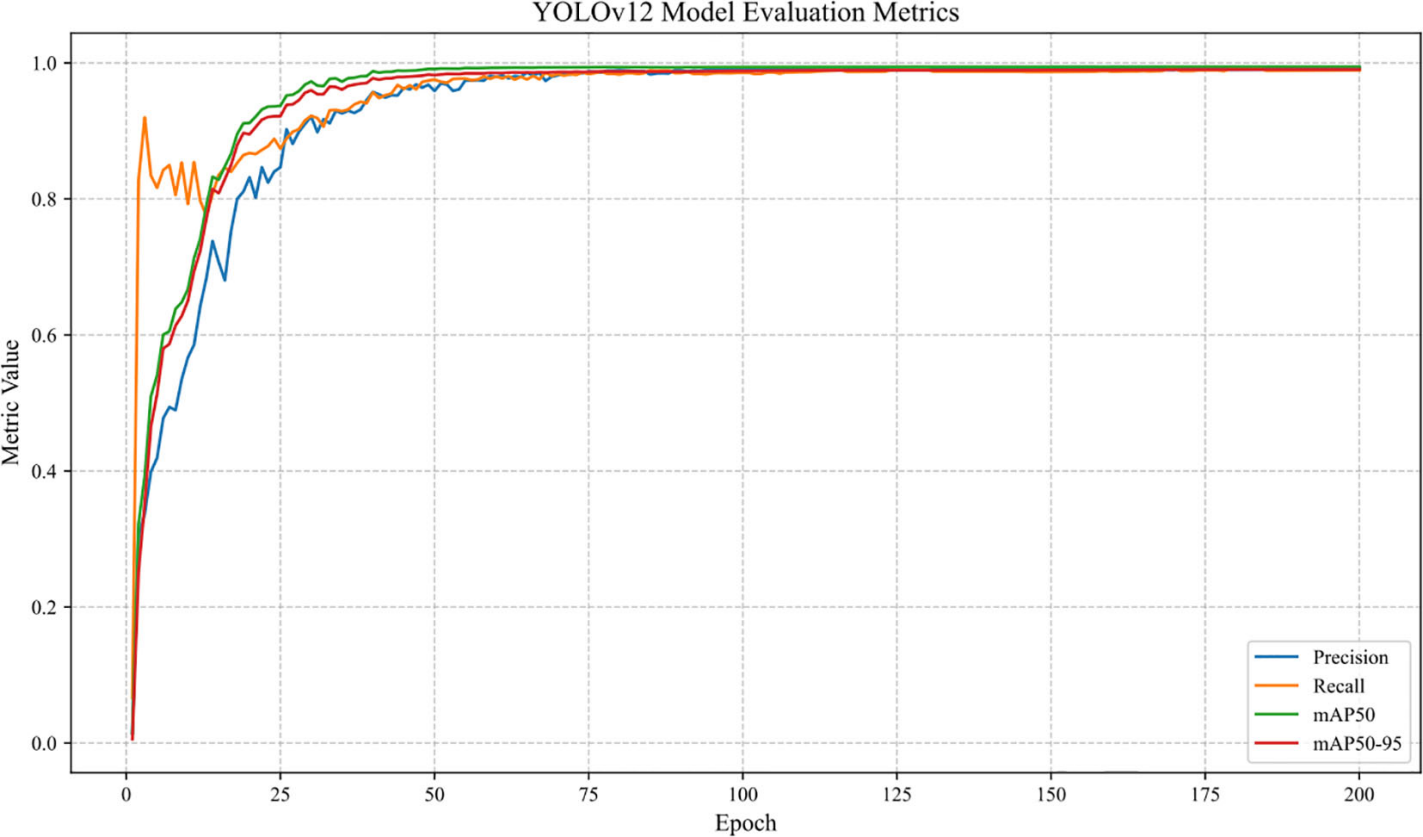
Changes in metrics during training of YOLOv12 baseline model.

The dynamic trends of the four core evaluation metrics—Precision, Recall, mAP50, and mAP50-95—for the YOLOv12 baseline model across 200 training epochs are shown in **Figure 12**.

The results can be interpreted from the following three aspects:

(1) Model convergence

All metrics increased rapidly during the initial training phase (0–50 epochs), indicating that the model gradually learned to capture the characteristic patterns of lychee anthracnose lesions. After epoch 50, the metrics stabilized: Precision, Recall, and mAP50 eventually reached plateaus within the range of 0.98–0.99, while mAP50–95 remained consistently around 0.99. These results demonstrate effective model convergence without significant overfitting.

(2) Stability of detection performance

The precision and recall were always at a high level (≥0.98), reflecting the model’s excellent prediction confidence and true target coverage of the spots, especially the consistency of identification of medium and heavy spots (Moderate, Severe).

The mAP50 (average accuracy at IoU=0.5) is stable at 0.994, and the mAP50-95 (average value of IoU from 0.5 to 0.95) is stable at 0.991, which verifies the robustness of the model under the requirements of different positioning accuracies, and it can maintain high performance even when the requirements of the accuracy of the bounding box position are increased (e.g., IoU=0.95).

(3) Baseline model validity

The result provides a baseline reference for subsequent lightweight improvements: the baseline model achieves 99.4% mAP50 on a dataset of 14, 576 images acquired by multiple devices, demonstrating the applicability of the YOLOv12 architecture to the task of grading lychee anthracnose. Meanwhile, the high stability of the metrics also lays the foundation for the subsequent comparison of the effects of introducing improved modules such as C3k2_Light, C2PSA, and wConv2D.

### 3.3 Ablation study

In order to study the impact of different modules on model performance, the comprehensive performance of different modules on the task of lychee disease classification was compared, involving detection precision indicators such as accuracy, recall, mAP50, mAP50-95, as well as model efficiency indicators such as the number of parameters and the amount of computation, which can clearly reflect the impact of the modules on the performance of the model when they are applied individually or in combination. The performance comparison is shown in **Table 5**.

**Table 5:** Comprehensive performance comparison of different modules in lychee disease grading task.

Model	Precision	Recall	mAP50	mAP50-95	Params	FLOPs
v12	0.993	0.989	0.994	0.991	2508929	5.8G
C3k2_Light	0.994	0.982	0.994	0.989	2498569	5.6G
C2PSA	0.989	0.986	0.994	0.989	2201345	5.6G
wConv2D	0.991	0.991	0.984	0.989	2508241	4.6G
C3k2_Light+C2PSA	0.99	0.986	0.993	0.989	2190985	5.4G
C2PSA+ wCovn	0.991	0.98	0.994	0.989	2200657	4.3G
C3k2_Light+wConv2D	0.989	0.989	0.986	0.989	2497881	4.3G
C3k2_Light+C2PSA+wConv2D	0.99	0.986	0.994	0.989	2190297	4.1G

**Figure 13:**
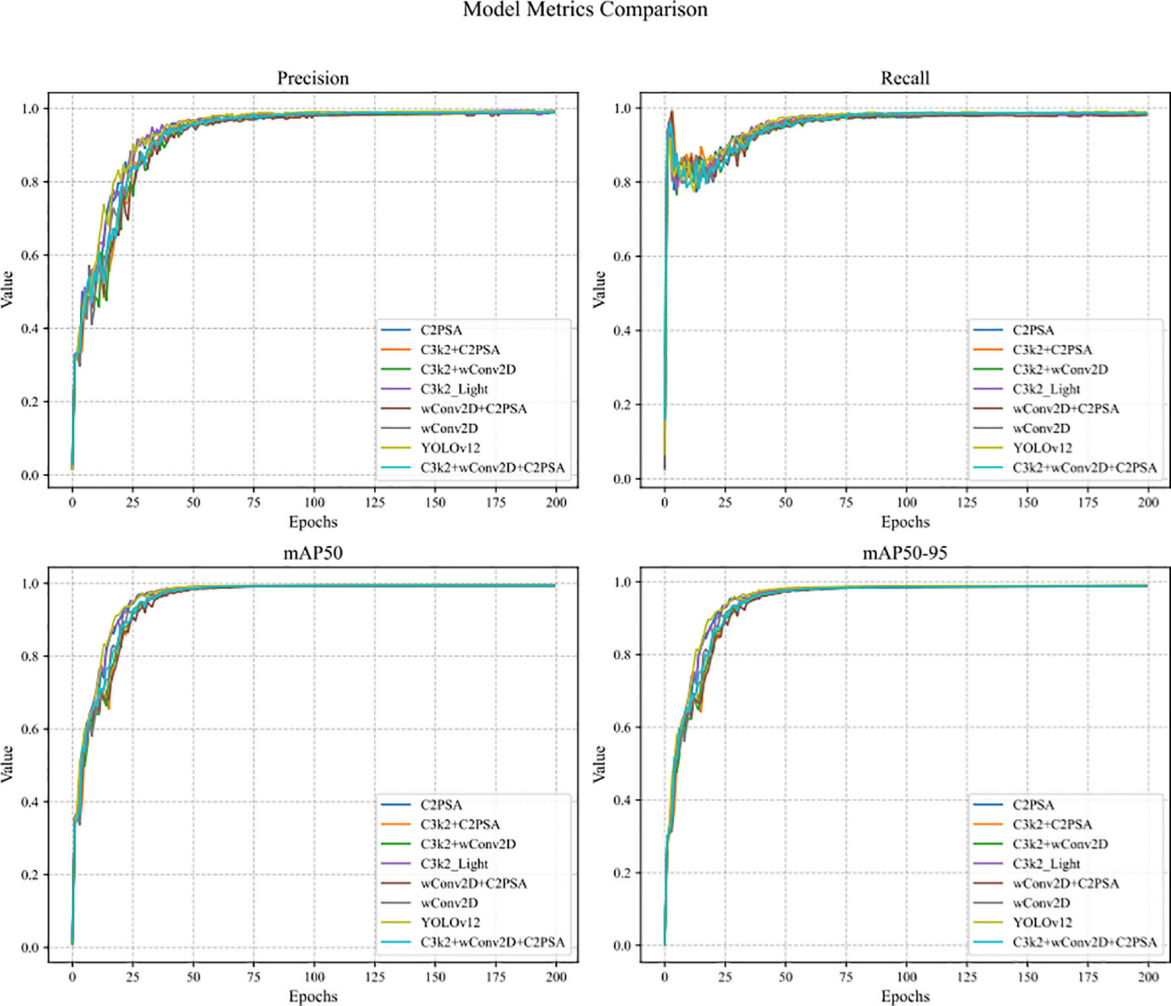
Trend of core metrics of each model with the number of training rounds.

**Figure 13** systematically illustrates the dynamic evolution of core performance metrics across the complete 200 training epochs for all models examined in this study. This encompasses the baseline YOLOv12 and its various improved variants, which integrate specialized modules such as C3k2_Light, C2PSA, and wConv2D. The figure employs a set of clear, comparative line charts to depict the complete trajectories of four key metrics—Precision, Recall, mAP50, and mAP50-95—throughout the training process. By examining these curves, one can trace the comprehensive learning pathway of each model, from initial state to final convergence. This allows for a detailed comparison of subtle differences between model configurations, including their convergence speed, training stability, and the ultimate performance plateau achieved. For instance, it becomes intuitive to identify whether introducing specific modules induces metric fluctuations or which combinations achieve performance saturation more rapidly. This dynamic, temporal perspective, which static numerical tables cannot adequately capture, provides crucial visual evidence for understanding how each architectural component influences the learning dynamics and overall robustness of the models.

**Figure 14:**
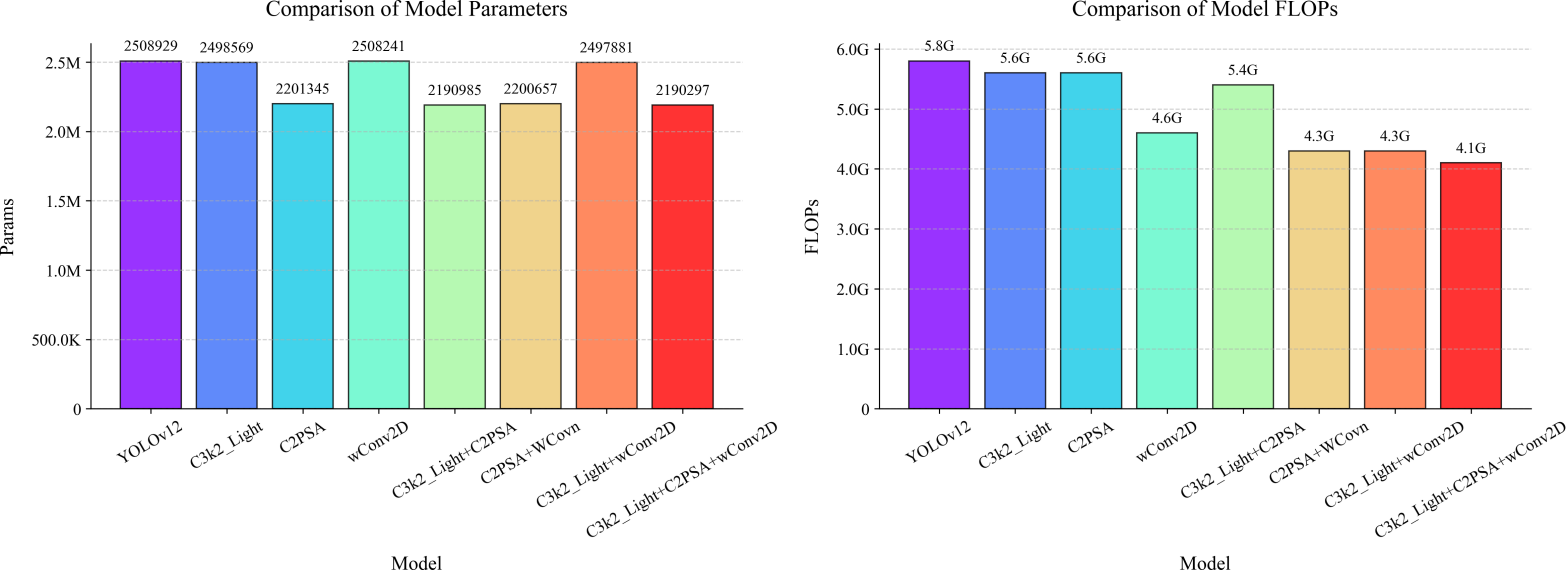
Comparison between the number of parameters and the amount of computation of each model.

**Figure 14** complements the ablation study by presenting a static analysis from the perspective of model complexity, thereby adding a critical efficiency dimension to the comparison. This figure utilizes two sets of juxtaposed bar charts to intuitively display two fundamental efficiency metrics for all compared models after training completion: the total parameter count (Params) and the computational cost in FLOPs. The height of each model’s bar directly corresponds to its computational burden and storage footprint, enabling an immediate visual comparison of the significant differences in lightweighting effectiveness. This includes comparisons between the baseline YOLOv12, various intermediate models incorporating single improvements, and the final comprehensive LycheeGuard-Lite model. This visualization method effectively delineates the specific contribution of each proposed module—such as the depthwise separable convolution in C3k2_Light, the structural optimization in C2PSA, and the dynamic weighting strategy in wConv2D—towards reducing both the parameter count and computational load. It thereby powerfully demonstrates how our co-design approach systematically and progressively enhances model efficiency and achieves notable lightweighting, all while maintaining high detection accuracy.

**Figure 13** contains four sub-figures, which show the dynamic changes of Precision, Recall, mAP50 and mAP50–95 of different models during the training process. The results show:

1. Precision and Recall: all models gradually converge after 50 training rounds, among which the three-module optimisation model (C3k2_Light+C2PSA+wConv2D), which integrates C3k2_Light, C2PSA and wConv2D, has a stable Precision of 0.990 and a Recall of 0.986.
2. mAP50 and mAP50-95: the mAP50 of each model is stable between 0.993-0.995, the mAP50 of the three-module optimisation model stays at 0.994, which is the same as the baseline; the mAP50–95 fluctuates around 0.989, which indicates that the improved model maintains good detection robustness under different IoU thresholds.

**Figure 14** contains two sub-figures, which show the number of parameters and computation amount of each model respectively. The results show:

1. **Number of parameters:** the baseline model parameter number of YOLOv12 is 2.51M, which is reduced to 2.20M (12.4% reduction) after introducing the C2PSA module, and the parameter number of the three-module combination model is further reduced to 2.19M, which is 12.8% less than that of the baseline, which reflects the synergistic effect of the parameter streamlining between C3k2_Light and C2PSA.
2. **Computational quantity:** The computational quantity of the baseline model is 5.8GFLOPs, which is reduced to 4.6GFLOPs (20.7% reduction) when the wConv2D module is used alone, and the computational quantity of the three-module combined model is further compressed to 4.1GFLOPs, which is 29.3% reduction compared with that of the baseline, verifying the effectiveness of the depth-separable convolution and the dynamically-weighted convolution on the reduction of the computational complexity.

Through the above analysis, we can further obtain the following conclusions:

**(1) C3k2_Light:** adopting depth-separable convolution to reconstruct the bottleneck structure, by reducing the number of parameter interactions in the convolution operation, the computation volume is reduced by 3.4% (from 5.8G to 5.6G) compared with the baseline, which provides the basic computational efficiency support for model lightweighting.

**(2) C2PSA:** Through the optimisation of the network structure, the parameter amount is reduced by 12.4% (2.51M → 2.20M), this parameter compression effect is the most prominent among all the lightweighting modules, which is the key support for model lightweighting. The Mild level recall in the separate module (0.974) is better than other lightweighting variants (e.g., 0.967 for C3k2_Light) but slightly lower than the baseline (0.978), suggesting that it compensates for the retention of tiny lesion features.The aforementioned phenomenon does not represent an absolute contradiction but rather reflects the differential performance of the C2PSA module across two dimensions: “offsetting the negative impacts of lightweighting” and “inherent risk of contextual information loss.”

From a mechanistic perspective, compared to the C3k2_Light module (which relies on depthwise separable convolutions to reduce parameters but tends to weaken fine-grained features of small lesions) and the wConv2D module (which dynamically weights based on pixel distance but often inadequately allocates weights to small-scale targets), the position-channel dual-path attention mechanism in C2PSA actively focuses on local features of lesions (e.g., edge textures and color differences in mild-stage light brown small spots). This mitigates the common issue of “feature dilution” in lightweight modules. Consequently, its recall rate for mild-stage lesions (0.974) is significantly higher than those of C3k2_Light (0.967) and wConv2D (0.965), demonstrating its “partial offsetting” effect on the drawbacks of lightweighting.

However, the local feature enhancement mechanism of C2PSA also has inherent limitations. If attention is excessively concentrated on already identified local lesion areas, it may overlook sporadically distributed tiny lesions in the image, resulting in a recall rate (0.974) that remains lower than that of the YOLOv12 baseline model (0.978). This quantitatively reflects the “loss of contextual information due to over-focusing.”

In summary, the “offsetting effect” of the C2PSA module is relative to other single lightweight modules and does not entirely eliminate the performance degradation caused by lightweighting. This explains why its recall rate is higher than other lightweight modules yet lower than the baseline.

**(3) wConv2D:** Through the dynamic convolutional optimisation strategy, feature extraction weights are adaptively adjusted based on pixel spatial distances to reduce ineffective feature computation, resulting in a sharp reduction of 20.7% in computation for the separate module compared to the baseline (from 5.8G to 4.6G), which is the core contributing module for the computation reduction.

**(4) The combination of all three modules achieves the optimal balance:** the computation volume is reduced by 29.3% to 4.1GFLOPs and the computation volume is reduced by 29.3% to 4.1GFLOPs, while the Mild recall rate is maintained at 97.2%, which achieves a highly efficient balance between lightweight (double compression of parameter and computation volume) and detection precision, and meets the dual requirements of real-time and precision of agricultural scenarios.

### 3.4 Grading performance analysis

In order to further explore the practical application effect of the models in the grading detection task of lychee anthracnose, this study evaluates the detection performance of the models in terms of accuracy, recall, mAP50 and mAP50–95 for the spots with different severity (Mild, Moderate, Severe), and the performance of the models at all levels of spot detection is shown in **Table 6**.

**Table 6:** Spot detection performance at various levels.

Model	Severity level	Precision	Recall	mAP50	mAP50-95
v12	Mild	0.994	0.978	0.994	0.989
	Moderate	0.987	0.993	0.994	0.99
	Severe	0.997	0.997	0.992	0.993
C3k2_Light	Mild	0.996	0.967	0.994	0.989
	Moderate	0.986	0.983	0.992	0.987
	Severe	0.999	0.995	0.995	0.992
C2PSA	Mild	0.987	0.974	0.994	0.986
	Moderate	0.982	0.988	0.994	0.989
	Severe	0.997	0.996	0.995	0.993
wConv2D	Mild	0.995	0.965	0.994	0.987
	Moderate	0.98	0.991	0.994	0.988
	Severe	0.998	0.996	0.995	0.993
C3k2_Light+C2PSA	Mild	0.989	0.972	0.994	0.987
	Moderate	0.984	0.989	0.991	0.987
	Severe	0.998	0.996	0.995	0.993
C2PSA+wConv2D	Mild	0.992	0.96	0.994	0.987
	Moderate	0.985	0.985	0.992	0.987
	Severe	0.995	0.996	0.995	0.992
C3k2_Light+wConv2D	Mild	0.989	0.971	0.994	0.987
	Moderate	0.983	0.99	0.993	0.988
	Severe	0.994	0.996	0.995	0.992
C3k2_Light+C2PSA+wConv2D	Mild	0.99	0.972	0.994	0.987
	Moderate	0.985	0.989	0.992	0.986
	Severe	0.995	0.996	0.995	0.993

**Figure 15:**
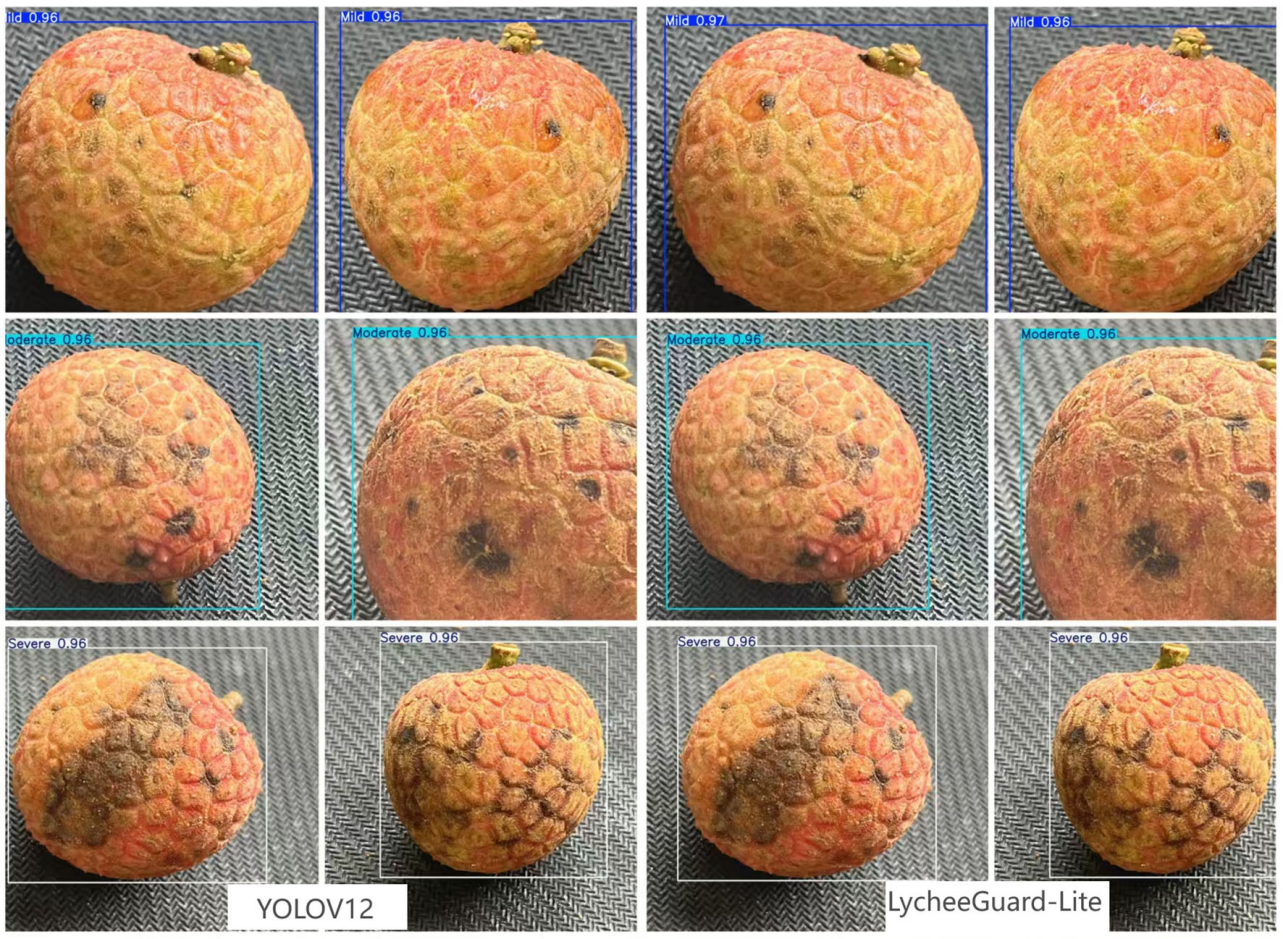
Comparison of YOLOv12 and LycheeGuard-Lite detection results.

**Figure 15** provides a direct visual comparison of detection results between the baseline YOLOv12 model and our proposed LycheeGuard-Lite model on representative lychee samples. The side-by-side visualization demonstrates both models’ performance in localizing anthracnose lesions and classifying severity levels (Mild, Moderate, Severe) under identical conditions. Critical examination reveals that LycheeGuard-Lite maintains precise bounding box regression for various lesion sizes while correctly assigning severity labels with high confidence scores. Particularly noteworthy is its preserved capability in detecting subtle Mild-stage lesions (≤8mm diameter) despite significant parameter reduction. The comparative visualization substantiates that our architectural improvements achieve competitive detection quality relative to the baseline, validating that the substantial computational efficiency gains (29.3% FLOPs reduction) do not compromise practical detection performance. This empirical evidence strengthens the credibility of LycheeGuard-Lite as a viable lightweight solution for real-world deployment.

**Table 6** shows in detail the performance of different models on three disease levels (Mild, Moderate, Severe) in the ablation experiment. **Figure 15** demonstrates the comparison of YOLOv12 and LycheeGuard-Lite detection visualisation results, which can be obtained from the experimental analysis:

**(1) Baseline model (v12):** shows very high precision on Severe level (Precision 0.997, Recall 0.997, mAP50 0.992), and the highest recall on Moderate level (0.993). the recall on Mild level (0.978) is relatively low, and it is the main challenge point for small target detection.

**(2) Single module impact:**

C3k2_Light: the recall of Mild level decreases significantly (0.978→0.967), but the precision of Severe level reaches the highest (0.999). The reduced computation is its core advantage.
**C2PSA:** while maintaining the mAP50 at Mild level (0.994), the recall (0.974) is slightly lower than the baseline (0.978), but better than other lightweight modules (e.g., 0.967 for C3k2_Light), which suggests that it compensates for feature retention of tiny lesions.
**wConv2D:** With the dynamic convolution strategy, the computation of the separate modules is reduced by 20.7% compared to the baseline, which provides a key support for the model lightweighting. the highest precision is achieved at the Mild level (0.995), but the recall decreases (0.978 → 0.965). High precision (0.998) and high recall (0.996) are maintained at the Severe level.

**(3) Combined modules and final model (Ours):**

Combined C3k2_Light and C2PSA: Mild level recall (0.972) slightly decreased compared to baseline (0.978), but better than C3k2_Light alone (0.967). The number of parameters was the lowest (2.19M).
Combination of C2PSA and wConv2D: lowest Mild level recall (0.960) but low computation (4.3G).
Combination C3k2_Light and wConv2D: Mild level recall (0.971) is close to the baseline and computationally low (4.3G).

**Figure 16:**
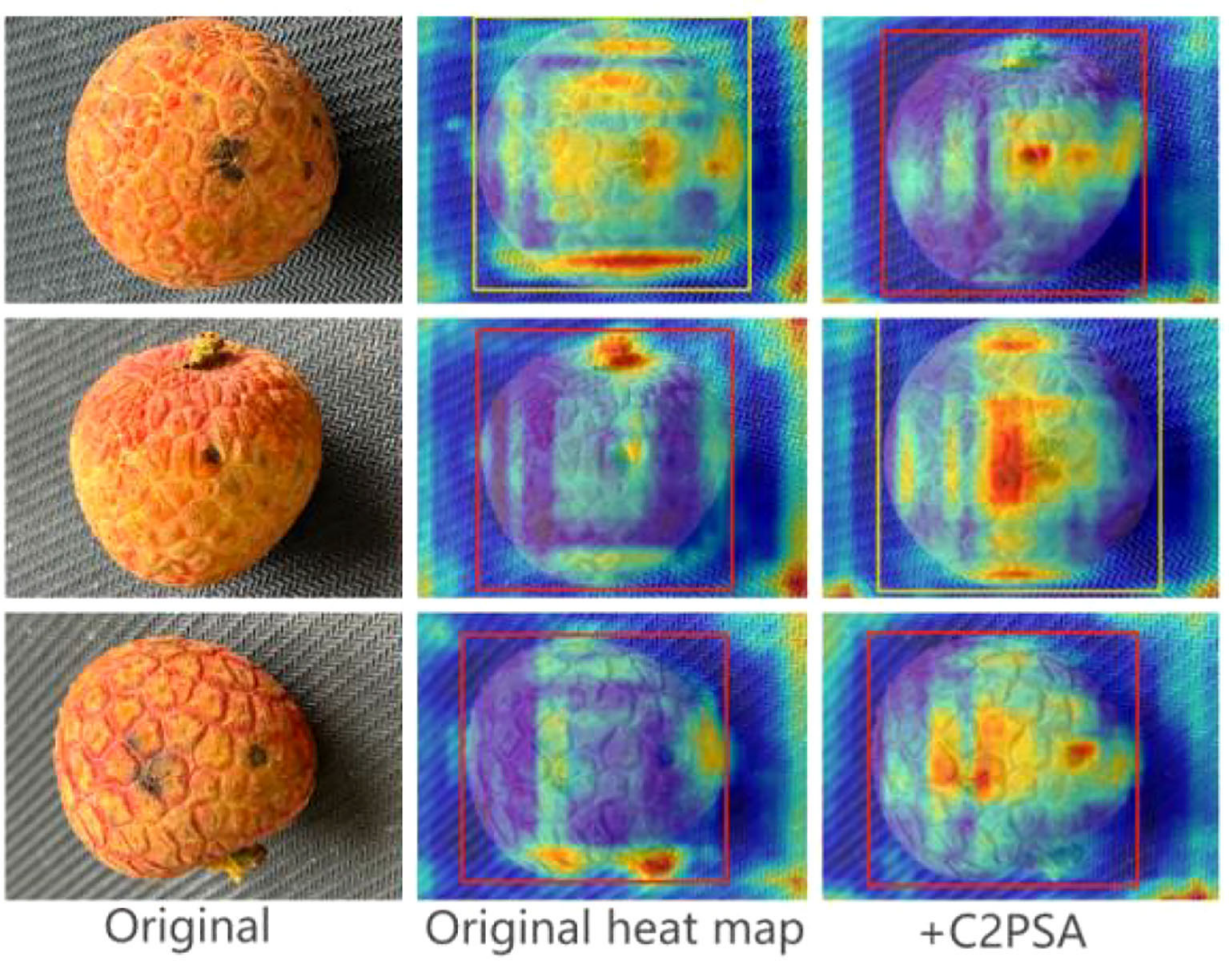
Comparison of heat map before and after adding C2PSA.

**The final three-module combined model (Ours):** achieved the highest recall (0.972) at the Mild level, significantly better than the other models except the baseline (0.978 at the baseline, but the final model computation/parameter count is much lower than the baseline). At the same time, very high performance was maintained at the Moderate and Severe levels (Precision 0.985/0.995, Recall 0.989/0.996, mAP50 0.992/0.995). Most importantly, the model achieves the lowest number of parameters (2.19M) and the lowest amount of computation (4.1GFLOPs) while maintaining high accuracy (mAP50 = 0.994), which perfectly balances accuracy and efficiency. **Figure 16** shows that while the model improvement achieves lightweighting, the accuracy is not significantly affected by the lightweighting.It should be noted that although the final model achieves a mild-level recall rate of 97.2%, outperforming all other ablation variants except the baseline, it remains lower than that of the YOLOv12 baseline (97.8%). The core reason lies in the “cumulative feature degradation” caused by the three improved modules:

Firstly, while the depthwise separable convolution in C3k2_Light reduces computational costs, its “channel-wise grouping” characteristic weakens the extraction of fine-grained features from small lesions (recall drops to 96.7% when used alone).

Secondly, although the local attention focus in C2PSA enhances certain lesion features, excessive localization leads to loss of global context, preventing complete capture of sporadically distributed small lesions (recall is 97.4% when used alone).

Thirdly, the pixel-distance-based weighting strategy in wConv2D inadequately allocates weights to small-scale targets, further diluting the feature signals of mild lesions (recall is 96.5% when used alone).

Although the synergy of these three modules achieves a balance between lightweight design and accuracy, a 0.6% recall gap persists. This gap primarily stems from the inherent contradiction between the “low-contrast, small-scale” nature of mild lesions and the “feature compression” design of lightweight modules.

## 4 Discussion

### 4.1 Deployment performance estimation and analysis

Although this study does not provide actual measured frame rates (FPS) on specific hardware, the deployment performance of the model can be reliably estimated based on its computational complexity (FLOPs) and parameter count (Params). The core lightweight improvements of the LycheeGuard-Lite model (2.19M parameters, 4.1 GFLOPs computational load) have laid a solid foundation for its efficient deployment.

Compared with mainstream lightweight models (as shown in **Table 7**), the computational load of our model is significantly lower than that of YOLOv8n (8.1G), YOLOv10n (6.5G), and YOLOv12n (5.8G). Based on general deployment experience for lightweight models, when processing images at a resolution of 416×416 on mainstream mobile computing platforms (such as Qualcomm Snapdragon 8 series or MediaTek Dimensity series chips), models with such computational loads typically achieve inference speeds of 30–50 FPS. On embedded devices with GPUs (e.g., NVIDIA Jetson Nano) or desktop-level GPUs, the frame rate can easily exceed 100 FPS.

**Table 7:** Performance comparison with lightweight models.

Models	Precision	Recall	mAP50	mAP50-95	Params	FLOPs	Memory usage	Training time
v8	0.988	0.988	0.994	0.991	3, 006, 233	8.1G	2.01G	15.7h
v10	0.979	0.983	0.993	0.984	2, 265, 753	6.5G	2.5G	13.6h
v11	0.987	0.985	0.994	0.989	2, 582, 737	6.3G	1.99G	15.6h
v12	0.993	0.989	0.994	0.991	2508929	5.8G	2.49G	15.6h
v13	0.986	0.992	0.994	0.99	2, 448, 480	6.1G	3.03G	17.4h
LycheeGuard-Lite	0.99	0.986	0.994	0.989	2, 190, 297	4.1G	2.18G	13.5h

### 4.2 Technical contributions and advantages

The core contribution of this work is the proposal and validation of a synergistic lightweight design paradigm tailored for “small-spot diseases” such as lychee anthracnose. The advantages demonstrated by the LycheeGuard-Lite model (C3k2_Light+C2PSA+wConv2D) can be distilled into the following conceptual insights:

(1) Excellent balance of lightness and accuracy:

The number of parameters (2.19M) is significantly lower than the baseline YOLOv12 (2.51M) and mainstream lightweight models such as YOLOv8n (3.2M).

The computation volume (4.1GFLOPs) is significantly lower than the baseline (5.8GFLOPs), indicating strong potential for efficient deployment on resource-constrained devices.While achieving significant lightweight, the key metric mAP50 remains consistent with the baseline (0.994), and the recall of the most challenging Mild grade small lesions is maintained at 97.2%, which is better than most ablation variants.This balance is not a coincidence but the direct result of our co-design strategy, where the parameter and computational reductions from C3k2_Light and wConv2D are actively compensated by the feature enhancement of C2PSA, effectively resolving the common trade-off between model size and accuracy.

(2) Novel feature enhancement mechanism counteracts lightweighting side effects:

The core value and novelty of the C2PSA mechanism lie in its targeted role to counteract the performance drop on small lesions—a typical side effect of aggressive lightweighting. When C3k2_Light caused the Mild recall to drop to 0.967, the introduction of C2PSA brought the combined model back up to 0.972 (still below the baseline of 0.978). The final model maintains the Mild recall at 97.2% with the constraints of 12.8% reduction in parameters and 29.3% reduction in computation through the synergy of the three modules.This demonstrates a novel lightweighting paradigm: instead of accepting performance degradation, we integrate a dedicated module to preserve task-critical features (small lesions) during complexity reduction.The heat map visualisation shows that C2PSA enhances the model’s focus on the local region of the lesion, but the quantitative results (**Table 5**) indicate that this mechanism fails to translate into an absolute increase in recall, possibly due to the over-focusing of attention resulting in a loss of global contextual information, as illustrated in the heat map in **Figure 16**.

(3) Enhanced robustness for agricultural environments through dynamic convolution:

The wConv2D module introduces a novel dynamic weighting strategy based on pixel spatial distance, which is distinct from static convolutions used in most models.

This design directly improves the stability of the model’s lesion localisation under simulated complex lighting (Front light, Side light, and Back light) conditions, with minimal fluctuations in mAP50 (0.994). This contribution is crucial for agricultural applications, as it emblights a degree of lighting invariance into the model, enhancing its practicality in real-world post-harvest scenarios where lighting control is often imperfect.

(4) Task-specific design addressing the gaps in agricultural vision:

A overarching novelty of this work is that all improvements are deeply tailored to the domain-specific characteristics of lychee anthracnose, rather than being generic lightweight tricks. The C2PSA module’s enhanced focus on local features is specifically designed for sub-8mm, low-contrast, light-brown spots that characterize the Mild grade. The wConv2D’s optimization is geared towards handling the specific lighting challenges (e.g., backlighting causing poor contrast) in sorting environments. This domain-specific co-design directly addresses the identified gaps in the literature regarding specialized models for “small-spot diseases” like lychee anthracnose, providing a more targeted solution than general-purpose detectors.

### 4.3 Comparison with other YOLO versions

In order to comprehensively evaluate the sophistication of LycheeGuard-Lite model in intelligent grading detection of lychee anthracnose, this study compared it with five mainstream versions of YOLOv8, YOLOv10, YOLOv11, YOLOv12 and YOLOv13 in a side-by-side manner. The experiment adopts a unified dataset of 14576 images, and uses a 7:2:1 divided dataset consistent with the above experiments, and takes Precision, Recall, Mean Average Precision (mAP50, mAP50-95), Parameter Counts (Params), Computational Volume (FLOPs), Memory Usage and Training Time as the core evaluation metrics. The performance comparison results of each model are shown in **Table 7**.

**Table 7** compares the performance of the final improved model of this study (LycheeGuard-Lite) with other mainstream YOLO versions on the lychee anthracnose grading task:

1. **mAP50:** YOLOv8, YOLOv11, YOLOv12, YOLOv13, and the present model all achieved a very high level (0.994). yOLOv10 is slightly lower (0.993).
2. **Recall:** YOLOv13 is the highest (0.992), YOLOv12 is the next highest (0.989), and the present model (0.986) is comparable to YOLOv8 (0.988) and YOLOv11 (0.985), and better than YOLOv10 (0.983).
3. **Lightweight:** this is the core advantage of this model. In terms of the number of parameters, this model (2.19M) is significantly lower than YOLOv8 (3.01M), YOLOv11 (2.58M), YOLOv12 (2.51M), YOLOv13 (2.45M), and only slightly higher than YOLOv10 (2.27M). In terms of the amount of computation (FLOPs), this model (4.1G) is substantially lower than all the compared models (YOLOv10: 6.5G, YOLOv11: 6.3G, YOLOv12: 5.8G, YOLOv13: 6.1G, YOLOv8: 8.1G), demonstrating excellent efficiency.
4. Memory Usage Efficiency: From the perspective of hardware compatibility, LycheeGuard-Lite exhibits a memory footprint of only 2.18M, significantly lower than YOLOv10 (2.5M), YOLOv12 (2.49M), and YOLOv13 (3.03M). Although slightly higher than YOLOv8 (2.01M) and YOLOv11 (1.99M), its advantages in parameter count and computational load (our model has fewer parameters than YOLOv8 and YOLOv11) demonstrate more efficient utilization of memory resources. This is particularly critical for embedded devices (e.g., agricultural sorting robots typically equipped with 2-4GB of VRAM), as it avoids detection interruptions caused by memory overflow.
5. Training Time Cost: In terms of model development efficiency, LycheeGuard-Lite achieves the shortest training time (13.5 hours), representing a 0.7% reduction compared to YOLOv10 (13.6 hours, which has a similar parameter count), a 13.5% reduction compared to the baseline YOLOv12 (15.6 hours), and a 22.4% reduction compared to the latest version YOLOv13 (17.4 hours). This advantage stems from its lightweight design (29.3% reduction in computational load), which reduces the time cost of iterative model testing and better aligns with the need for rapid algorithm optimization in agricultural scenarios.

**Comprehensive evaluation:**LycheeGuard-Lite achieves the lowest computational complexity (4.1G FLOPs) and an extremely low parameter count (2.19M), while maintaining superior memory efficiency (2.18MB) and shorter training time (13.5 hours)—all without compromising high detection accuracy (mAP50 = 0.994) and strong recall (0.986), comparable to state-of-the-art YOLO versions (v12, v13). These four key advantages not only lower the hardware barriers for mobile/embedded deployment (compatibility with low-VRAM devices) but also reduce model development time costs, making it the optimal solution for real-time grading of lychee anthracnose.Although the LycheeGuard-Lite model has achieved good results, there are still some limitations, which also point out the direction for future research: in terms of scene coverage, the current model focuses on single-fruit static image detection, and does not cover the fruit overlapping scenes that may occur in the actual production line; in terms of environmental robustness, the model training and testing are mainly based on indoor controllable lighting conditions, although the data augmented to In terms of environmental robustness, the model training and testing are mainly based on indoor controlled lighting conditions, although enhanced by data to simulate different lighting, but not systematically tested under extreme weather conditions, such as rain, fog, strong direct sunlight or severe shade; and in terms of the performance of the small targets, although the Mild level of anthracnose lychee recall (0.972) has already reached a higher level, there is still room for improvement compared with the Moderate and Severe levels, especially for very small or low colour contrast spots.

### 4.4 Advantages and limitations

This study successfully demonstrates the potential of a co-designed lightweight model for agricultural disease grading, yet its findings must be interpreted within the context of its specific scope and inherent design trade-offs.

The primary advantage of LycheeGuard-Lite is its validated efficacy in balancing competing objectives. It provides empirical evidence that a carefully structured lightweight model can achieve near-state-of-the-art accuracy for lychee anthracnose grading while drastically reducing computational demands. Furthermore, the proactive use of the C2PSA feature enhancement mechanism to partially offset the small-lesion performance penalty associated with lightweighting represents a constructive strategy for domain-specific model compression.

However, the model’s advantages are achieved within certain limitations. Most notably, the recall for Mild-grade lesions, while robust among lightweight models, remains 0.6% lower than the more computationally intensive YOLOv12 baseline. This reflects an inherent trade-off in lightweight design: achieving efficiency can come at a minor cost in sensitivity to the most subtle features. Additionally, the model’s validation has been conducted primarily under controlled, single-fruit conditions. Its performance in more complex, real-world environments—such as handling occluded fruits on a high-speed sorting line or operating under extreme, unconstrained outdoor lighting—remains to be fully validated and represents the current boundary of its generalizability.

These limitations naturally outline a clear path for future work. Efforts will focus on exploring advanced attention mechanisms to close the performance gap on small lesions without compromising efficiency. Concurrently, expanding the training dataset to include diverse field conditions and fruit arrangements will be crucial for enhancing the model’s robustness and practical applicability in fully automated agricultural systems.

## 5 Conclusion

In this study, a lightweight and improved YOLOv12 model, LycheeGuard-Lite, oriented towards fast and non-destructive grading of lychee anthracnose was proposed and validated.The key novelty of this work lies in the holistic integration of three lightweight enhancements: C3k2_Light for parameter reduction, C2PSA for feature retention in small lesions, and wConv2D for computational efficiency. Unlike existing models that focus solely on accuracy or lightweight design, LycheeGuard-Lite achieves a synergistic balance, laying a solid algorithmic foundation for future deployment on resource-constrained devices.The main technological innovations include:

1. Designing the C3k2_Light module to reconstruct the bottleneck structure using deeply separable convolution to effectively reduce the model complexity;
2. Introducing the C2PSA position-channel two-way attention mechanism, which achieves a significant reduction in the number of parameters through network structure optimisation (the number of parameters of the separate module is reduced by 12.4% from the baseline), and at the same time has an auxiliary effect on feature retention, and assists in maintaining the Mild-level recall of 97.2% (a decrease of 0.6% from the baseline) by synergising with other modules in the final model;
3. The introduction of wConv2D weighted convolution, which adaptively adjusts the feature extraction weights according to the pixel spatial distance, significantly reduces the computation (20.7% reduction from baseline for the separate modules).

On a rigorous test set containing 14, 576 multi-device captured lychee images, the LycheeGuard-Lite model significantly reduces the model complexity (2.19M parametric quantities, 12.8% reduction from the baseline; 4.1GFLOPs of computation, 29.3% reduction from the baseline) while maintaining a high detection accuracy (mAP50 = 99.4%). Ablation experiments and comparative analyses confirmed:

1. The model has stable and high-precision recognition ability for three classes of lychee anthracnose (especially Mild grade small spots), and the Mild grade recall rate is maintained at 97.2%.
2. The lightweight design makes its computational efficiency significantly better than mainstream YOLO versions (v8, v10, v11, v12, v13), effectively adapting to the deployment needs of mobile or embedded devices.
3. The data enhancement strategy combined with multi-angle light simulation guarantees the robustness of the model in the actual variable post-processing light environment.

Although C2PSA demonstrated local feature enhancement for small spots in thermograms (**Figure 13**), its Mild recall (0.974) was still lower than the baseline, suggesting that the attention mechanism may have missed some spots by overfocusing on local regions and ignoring the global context.

LycheeGuard-Lite provides reliable and lightweight core algorithm support for the development of low-cost and portable post-harvest intelligent grading equipment for lychee, which is expected to enhance the grading efficiency and standardisation of the lychee industry, and to promote the intelligent upgrading of the industry. Future work will focus on:

1. **Fine grading output:** develop precise quantification algorithms for lesion area, integrate them into the detection head or as a post-processing module, and output the precise percentage of lesions occupying the fruit surface, so as to provide a more detailed basis for grading.
2. **Expanding application scenarios:** research on instance segmentation or detection techniques in fruit overlapping scenarios; development of model variants suitable for *in-situ* detection in the field, combined with proximal sensing technologies (e.g., hand-held devices, orchard robots) and light invariant feature extraction methods to realise pre-harvest disease monitoring.

## Data Availability

The datasets presented in this study can be found in online repositories. The names of the repository/repositories and accession number(s) can be found below: https://doi.org/10.57760/sciencedb.28773.
